# Novel magnetic bimetallic AuCu catalyst for reduction of nitroarenes and degradation of organic dyes

**DOI:** 10.1038/s41598-024-56559-4

**Published:** 2024-03-11

**Authors:** Mohammad Gholinejad, Saba Bashirimousavi, José M. Sansano

**Affiliations:** 1 Department of Chemistry, Institute for Advanced Studies in Basic Sciences (IASBS), Gavazang, P. O. Box 45195‐1159, Zanjan, 45137‐66731 Iran; 2 Research Center for Basic Sciences & Modern Technologies (RBST), Institute for Advanced Studies in Basic Sciences (IASBS), Zanjan, 45137‐66731 Iran; 3 Departamento de Química Orgánica, Instituto de Síntesis Orgánica, and Centro de Innovación en Química Avanzada (ORFEO-CINQA), Universidad de Alicante, 03690 Alicante, Spain

**Keywords:** Bimetallic, Magnetic, AuCu, Water dispersible, Reduction, Environmental sciences, Chemistry, Nanoscience and technology

## Abstract

Herein, core–shell magnetic nanoparticles are modified with imidazolium-tagged phosphine and propylene glycol moieties and used for the stabilization of bimetallic AuCu nanoparticles. The structure and morphology of the prepared material are identified with SEM, TEM, XRD, XPS, atomic absorption spectroscopy, Fourier translation infrared spectroscopy, and a vibrating sample magnetometer. This hydrophilic magnetic bimetallic catalyst is applied in the reduction of toxic nitroarenes and reductive degradation of hazardous organic dyes such as methyl orange (MO), methyl red (MR), and rhodamine B (RhB), as well as in the degradation of tetracycline (TC). This magnetic AuCu catalyst indicated superior activity in all three mentioned reactions in comparison with its single metal Au and Cu analogs. This catalyst is recycled for 17 consecutive runs in the reduction of 4-nitrophenol to 4-aminophenol without a significant decrease in catalytic activity and recycled catalyst is characterized.

## Introduction

Magnetic catalysts with high surface-area-to-volume ratio, super magnetism, easy separation, high mechanical and thermal stability, simple synthesis method, nontoxicity, and biocompatibility are considered as promising materials for designing heterogeneous catalysts^1^. Among different magnetic compounds, iron oxide nanoparticles (Fe_3_O_4_⋅NPs) with unique combination of properties, were widely utilized as support for stabilization of different transition metal catalysts^2–6^. For achieving magnetic catalysts with the desired activity and properties such as high water dispersibility, these compounds can modify with various ligands, polymers, and ionic liquids. Ionic liquids are molten salts that receive special attention due to their unique chemical and physical features in various fields, as well as the design of the catalysts^7,8^. However, high price and difficulty of their synthesis encourage chemists to support them on the surface of solid supports. This approach leads to a diminution in the amount of used IL and allowing the reaction on the thin IL layer on the support surface^1,9^.

Gold-based catalysts have received much attention over the past two decades. Gold is a productive element and very attractive in metal coordination area and organometallic chemistry and is extensively effective as a homogeneous or heterogeneous catalyst^10–12^. Despite the efficiency and scarce catalytic application on an industrial scale. One effective way to solve this problem is to combine gold with cheap and available metals such as copper, nickel, and cobalt to create gold based bimetallic catalysts^13,14^. Due to new electronic and physical properties and achieving synergetic effect between two combined metals, usually activity of bimetallic catalysts can be promoted compared to the single metal catalysts^15,16^. Along this line, very recently, different efforts have been paid to design and preparation of bimetallic gold catalysts such as NiAu, CoAu, PdAu, and CuAu in variety of organic reactions^17–21^.

Recently, the rapid growth of industrialization has led to environmental pollution caused by toxic compounds^22^. Organic dyes are classified as serious contaminants that many industries, such as rubber processing, food processing, textile, cosmetics, leather, printing, and pharmaceuticals, use in their manufactures. In addition, some portions of these dyes are released into sewage producing harmful effects to humans, animals, and all living organisms^23,24^. Adsorption, coagulation, oxidation, and membrane separation are some of the practical methods for removing dyes from industrial effluents^25^. Tetracycline (TC) is another damaging contaminant, which is broadly utilized as antibiotic in the bacterial infections treatment, agriculture, and fish farming. Released TC can be stay in the environment for a long time due to its high chemical stability, leading to increase the resistance of microorganisms, disruption of photosynthesis in aquatic organisms, and acute toxicity to humans^26–28^. Advanced oxidation and adsorption are the two main and accepted methods for eliminating tetracycline from wastewater^29^.

In addition, nitroaromatic compounds, especially nitrophenols, are among the large industrial wastes and pose a high risk to human and animal health due to their mutagenic and carcinogenic potential^30^. Numerous methods have been used to decompose or convert nitroarenes, with the catalytic reduction of them being the most suitable approach. Interestingly, the produced amines are useful building blocks for the synthesis of antioxidants, drugs, pharmaceuticals, agrochemicals, dyes, pesticides, and polymers^31^. Among different efficient, economical, and environmentally friendly processes for removing pollutants from the environment, much attention has been paid to the employment of heterogeneous catalysts with superior activity and good recyclability using conventional methods such as centrifugation and filtration, which are time-consuming, wasteful, and industrially inefficient^32^.

Herein, we report a novel phosphine, ionic liquid, and diol-modified magnetic nanoparticles supported AuCu species, Fe_3_O_4_@Phos-IL-AuCu, as an extremely water-dispersible and magnetically recyclable catalyst with high efficiency in the reduction of nitroarenes, organic dyes, and degradation of tetracycline.

## Result and discussion

**Scheme 1:**
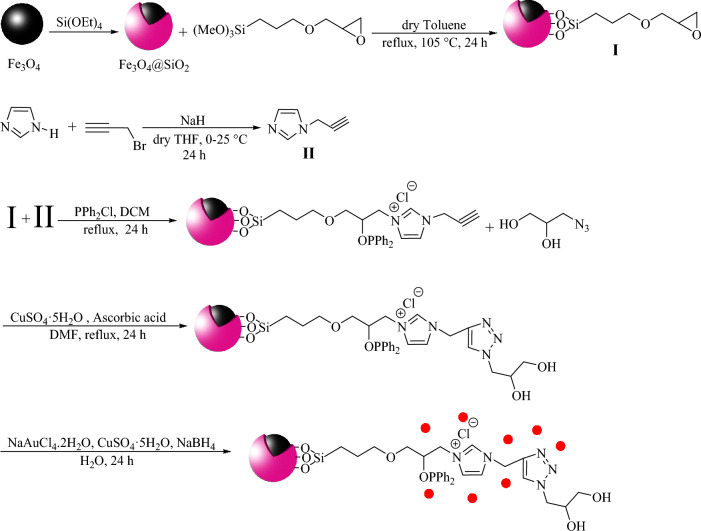
Preparation of Fe_3_O_4_@Phos-IL-AuCu.

The complete procedure for the preparation of the titled bimetallic catalyst is detailed in Scheme 1. The freshly prepared Fe_3_O_4_ nanoparticles, obtained from the reaction of FeCl_2_⋅4H_2_O and FeCl_3_⋅6H_2_O, were treated with Si(OEt)_4_ to afford Fe_3_O_4_@SiO_2_ core-shell. Next, (3-glycidyloxypropyl)trimethoxysilane was used to introduce the epoxy group (I). Then, the synthesized 1-(2-propynyl)-1H-imidazole (II) and chlorodiphenylphosphine were allowed to react with epoxy-functionalized magnetic nanoparticles(I) to achieve phosphorous and IL-modified MNPs. The alkyne group was then subjected to CuAAC reaction to introduce diol group. Finally, modified MNPs were treated with Au and Cu salts followed with reaction with NaBH_4_ to give AuCu supported on modified MNPs. This material referred to as Fe_3_O_4_@Phos-IL-AuCu NPs throughout of this article.

**Figure 1:**
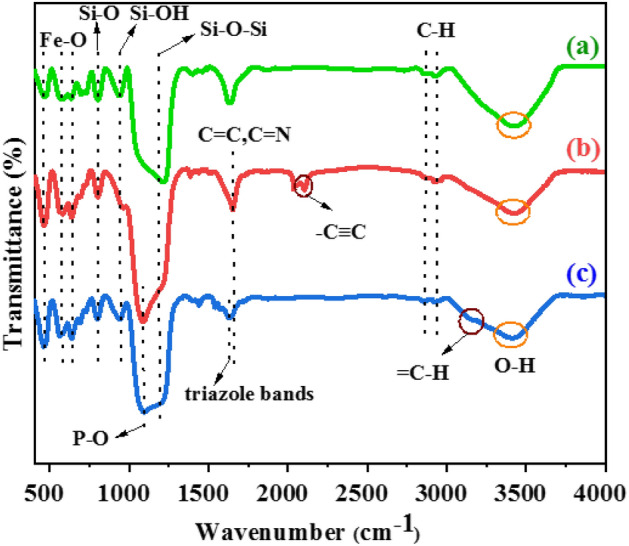
FT-IR spectra of preparation of (**a**) Fe_3_O_4_@SiO_2_@epoxide, (**b**) Fe_3_O_4_@C_2_H_4_OPPh_2_@1-(2-propynyl)-1H-imidazole, and (**c**) Fe_3_O_4_@Phos-IL.

The contents of Au and Cu in the Fe_3_O_4_@Phos-IL-AuCu were quantified by ICP to be 0.03 and 0.07 mmol/g, respectively. Every stage of the preparation of Fe_3_O_4_@Phos-IL-AuCu is analyzed by FT-IR spectroscopy, as shown in (Fig. 1). Peaks located at 461 cm^−1^, 570 cm^−1^, and 638 cm^−1^ corresponded to the Fe–O vibrations. Signals positioned at 800 cm^−1^, 954 cm^−1^, and 1119 cm^−1^ are derived from the Si–O, Si–OH, and Si–O–Si vibrations, respectively^33,34^. Broad bands centered at 2867 cm^−1^ and 2927 cm^−1^ were caused by C–H stretching vibrations^35^. The spectra of Fe_3_O_4_@SiO_2_–OPPh_2_–propargylimidazole and Fe_3_O_4_@Phos-IL compounds reveal that the peak at 1650 cm^−1^ is related to the C=N and C=C bonds of imidazole^36–38^. However, this peak overlaps with the bending vibration of absorbed water molecules. Additionally, the present peak at 1071 cm^−1^ in the spectrum of these compounds is due to the vibrations of the P–O bond, which is overlapping with the peak corresponding to the stretching vibration of the Si–O–Si bond^39^. In addition, the peak at 2120 cm^−1^ in the spectrum of Fe_3_O_4_@SiO_2_–OPPh_2_-propargylimidazole is ascribed to the existence of the terminal alkyne group^40^. The disappearance of this peak in the final stage of the compound (Fe_3_O_4_@Phos-IL) signifies the successful completion of the CuAAC 1,3-dipolar reaction occurred. In the Fe_3_O_4_@Phos-IL spectrum, the peak at 3138 cm^−1^ is caused by the stretching vibration of the C–H bond of the triazole moiety, while the peak at 1636 cm^−1^ is the results from overlapping bending vibrations of water, as well as C=C and C=N vibrations of imidazolium^41,42^. In all cases, the wide band centered at 3400 cm^−1^ is associated to O–H stretching vibration^43^ (Fig. 1).

**Figure 2:**
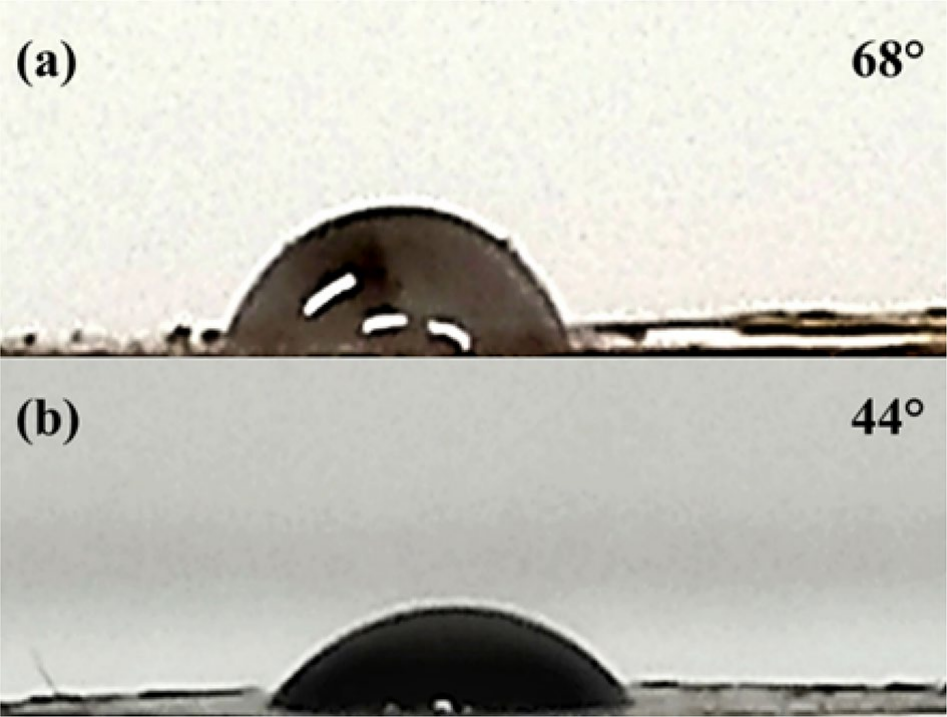
Water droplet on the prepared pellets from (**a**) Fe_3_O_4_@C_2_H_4_OPPh_2_@1-(2-propynyl)-1H-imidazole and (**b**) Fe_3_O_4_@Phos-IL.

The ionic liquid increased the hydrophilic nature of the catalyst. This effect was studied by calculating the contact angle of the Fe_3_O_4_@C_2_H_4_OPPh_2_@1-(2-propynyl)-1H-imidazole, and Fe_3_O_4_@Phos-IL aggregates. Results showed that contact angle of Fe_3_O_4_@Phos-IL is lower than the contact angle observed for Fe_3_O_4_@C_2_H_4_OPPh_2_@1-(2-propynyl)-1H-imidazole, confirming the impressive effect of ionic liquid in increasing hydrophilicity (Fig. 2).

**Figure 3:**
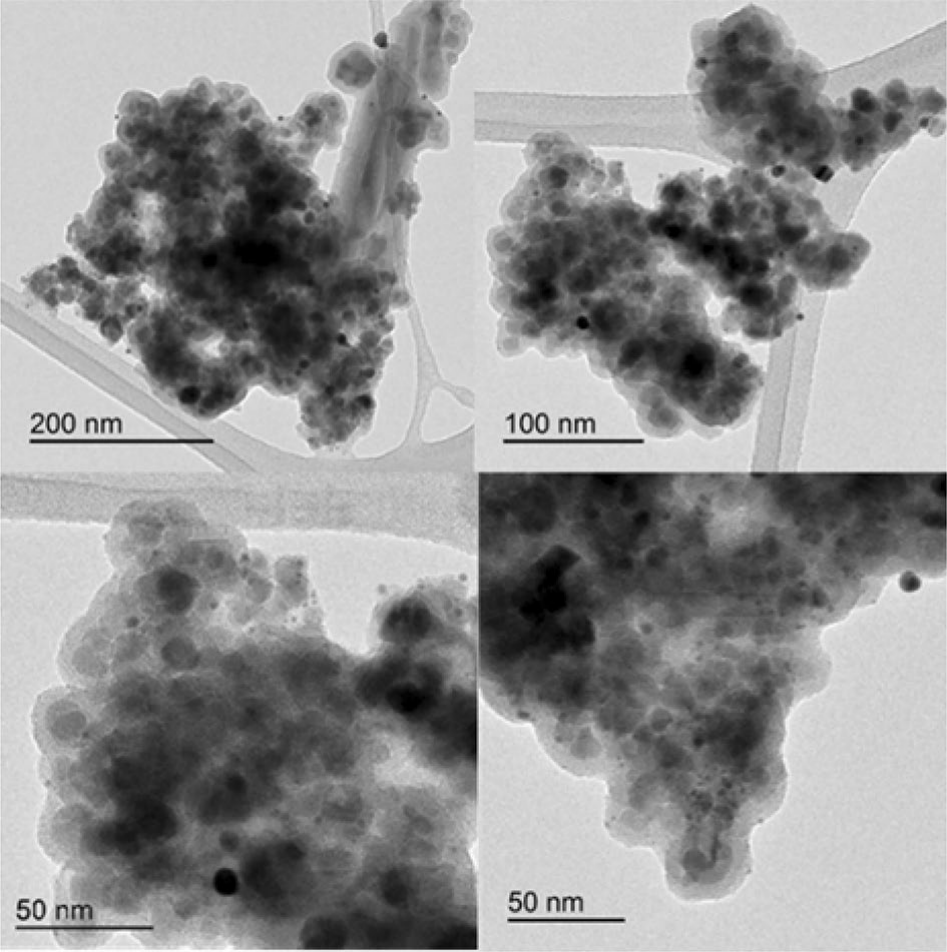
TEM images of Fe_3_O_4_@Phos-IL-AuCu.

The results of transmission electron microscopy (TEM) analysis of Fe_3_O_4_@Phos-IL-AuCu NPs samples revealed a distinct core-shell structure, with evidence of the presence of AuCu nanoparticles on the surface of the substrate. The determined average size of the AuCu NPs is 1.1 nm (see Fig. S1, Fig. 3).

**Figure 4:**
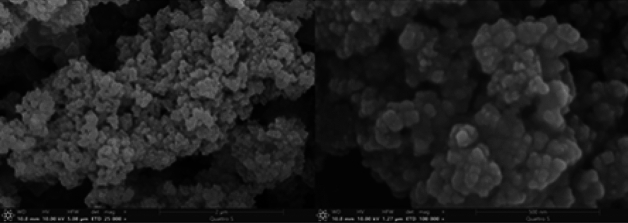
SEM images of Fe_3_O_4_@Phos-IL-AuCu.

The scanning electron microscopy (SEM) images revealed the formation of Fe_3_O_4_@Phos-IL-AuCu NPs nanoparticles with a uniform distribution, good dispersion, and near spherical morphology (Fig. 4).

**Figure 5:**
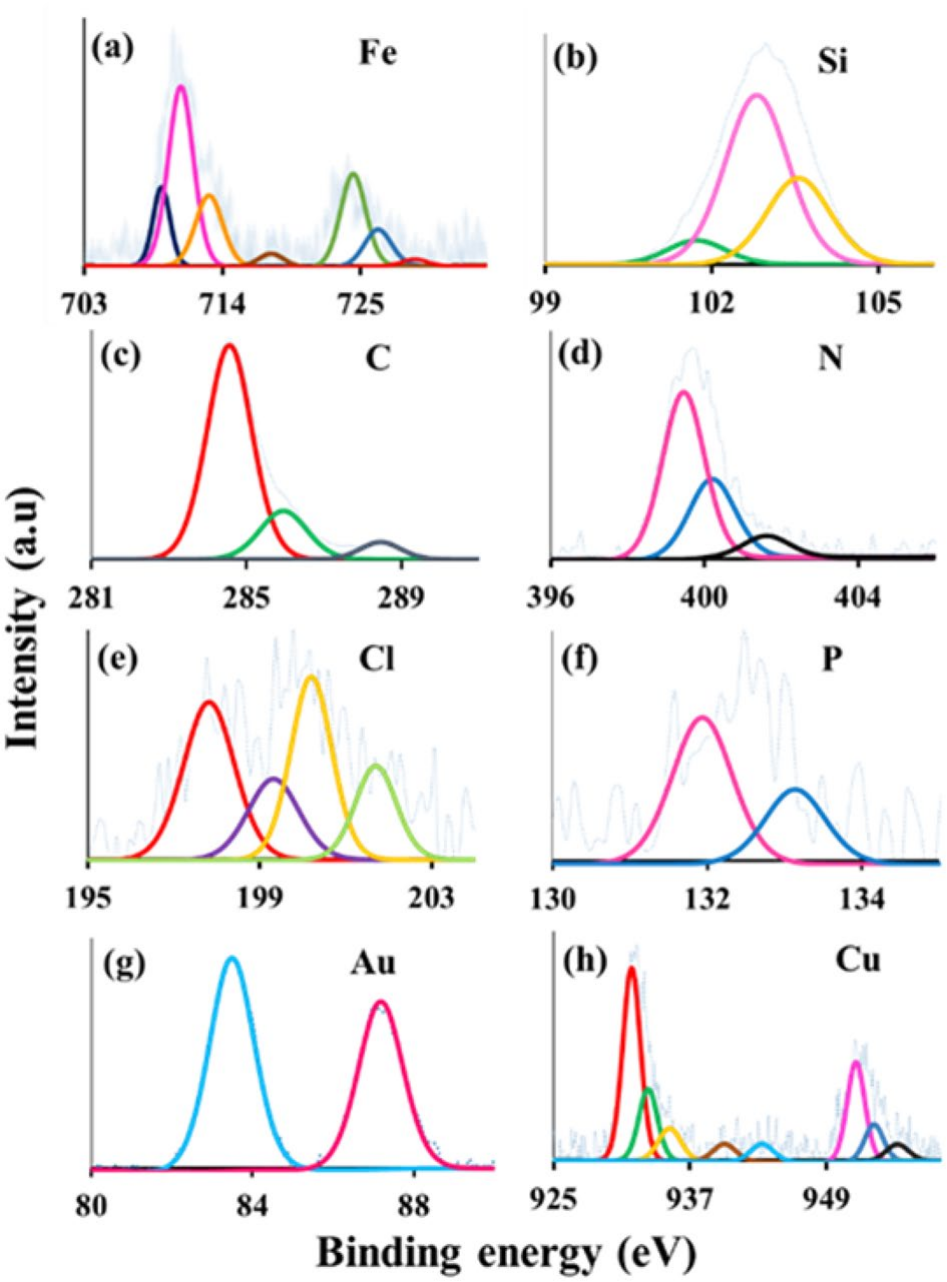
XPS spectra of Fe_3_O_4_@Phos-IL-AuCu in Fe, Si, C, N, Cl, P, Au, and Cu regions.

X-ray photoelectron spectroscopy (XPS) of Fe_3_O_4_@Phos-IL-AuCu in Fe, Si, C, N, Cl, P, Au, and Cu areas was analyzed (Fig. 5). The XPS plot of the Fe 2p region showed peaks at 709, 710, 712.5, and 716 eV, which correspond to Fe 2p3/2. Also, in this spectrum, the peaks in the regions of 723.9, 725.5 and 728.5 eV corresponded to Fe 2p1/2 (Fig. 5a)^44–46^. In the Si 2p spectroscopy, the peaks at 101.6, 102.8, and 103.5 eV corresponded to Si–C, Si–O–C, and O–Si–O, respectively (Fig. 5b)^47,48^. XPS analysis of C 1s plot showed peaks located at 284.8, 286, and 288.5 eV which were ascribed to C–C, C–OH, and N–C=N, individually (Fig. 5c)^49,50^. In the N 1s spectroscopy, the peak at 399.5 eV was associated to the C–N bond, the 400 eV peak was attributed to the C=N bond, and the peak at 401.6 eV in this region was associated to the NR_3_^+^ species in imidazolium (Fig. 5d)^51–53^. In the Cl 2p spectral analysis, the peaks at 197.5, 199.4, and 201.7 eV were attributed to the Cl 2p3/2, while the peak at 200.4 eV was associated with the Cl 2p5/2 (Fig. 5e)^54–57^. XPS of phosphorus revealed two peaks in the 2p region, located at 131.2 and 133.4 eV, corresponding to the bonding states of P–C and P–O, respectively (Fig. 5f)^58,59^. Additionally, the XPS plot of the gold 4f area revealed two peaks located at 83.5 and 87.5 eV, both of them conforming to the Au(0) (Fig. 5g)^60^. Finally, the XPS analysis was performed on the Cu 2p area, revealing two signals at 931.5 and 932 eV, corresponding to Cu_2_O. The peak observed at 933.5 eV was attributed to Cu 2p3/2, while the peaks at 953.4 and 955 eV were assigned to Cu 2p1/2 in CuO. Additionally, smaller peaks in the regions of 935, 940, and 943 eV were attributed to metallic copper(II), copper(II) oxide and copper(II) hydroxide (Fig. 5h)^61–63^.

**Figure 6:**
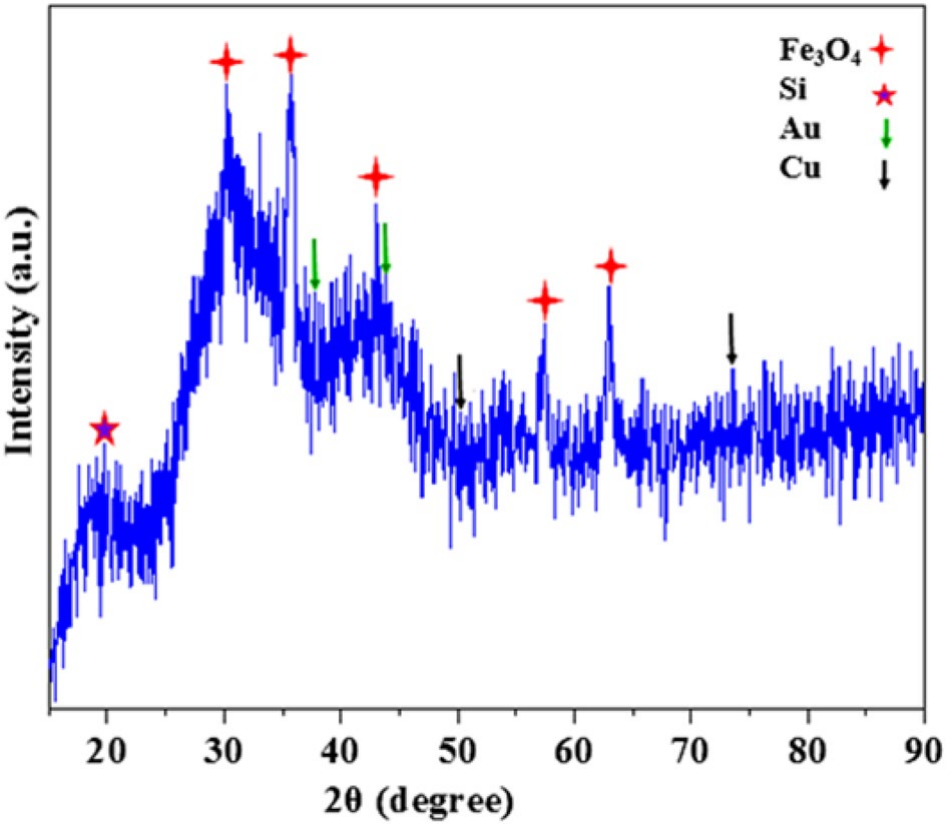
XRD pattern of Fe_3_O_4_@Phos-IL-AuCu.

The X-ray diffraction analysis of the Fe_3_O_4_@Phos-IL-AuCu NPs catalyst demonstrated the existence of magnetic Fe_3_O_4_ nanoparticles, evidenced by the Bragg reflections at 2θ = 30.1°, 35.5°, 43.1°, 57.2°, and 62.8° ^64,65^. The reflections at 2θ = 43.85° and 37.58° corresponded to the gold nanoparticles in the bimetallic gold-copper structure, while the reflections at 2θ = 50.0° and 73.8° corresponded to the copper nanoparticles^66–69^. Additionally, a wide band around 2θ = 22.0 was related to SiO_2_ (Fig. 6).

**Figure 7:**
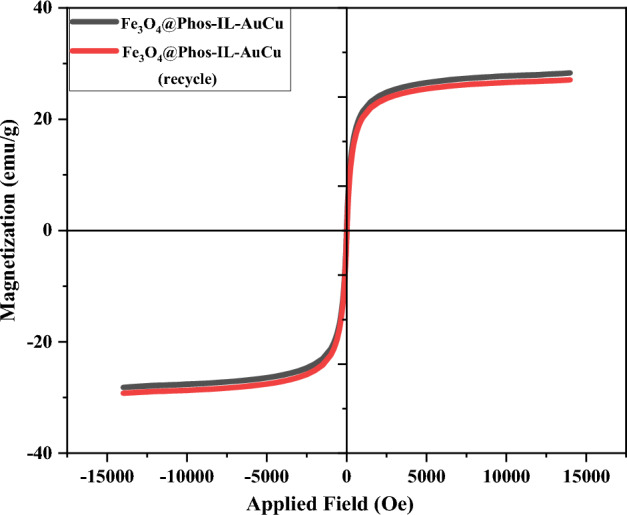
Magnetization curves for Fe_3_O_4_@Phos-IL-AuCu and recycled Fe_3_O_4_@Phos-IL-AuCu.

The superparamagnetic behavior of Fe_3_O_4_@Phos-IL-AuCu NPs was studied by vibrating-sample magnetometer (VSM) in which the results indicated zero coercivity together with remanence on the magnetization-loop, proving the superparamagnetic nature of the material and the possibility of easy separation by an external magnetic field (Fig. 7).

After the characterization of Fe_3_O_4_@Phos-IL-AuCu NPs, its catalytic activity was initially assessed in the reduction of nitro aromatic compounds. The reduction of the starting compound, 4-chloronitrobenzene, was selected as the reference reaction and various factors like type of reductant, solvent, catalyst loading (mol%), and reaction time were analyzed (Table 1). Initial results indicated that a 50% GC yield was obtained by using the catalyst with 0.06 mol% Au, 0.16 mol% Cu and using NaBH_4_ (2 mmol) in H_2_O during 2 h at room temperature (Table 1, entry 1). Results showed that with the enhancement of the catalyst amount to Au (0.1 mol%) and Cu (0.24 mol%), a 65% yield of the desired amine was obtained (Table 1, entry 2), while with higher amounts of Au (0.15 mol%) and Cu (0.35 mol%), a quantitative yield was achieved (Table 1, entry 3). Additionally, the yield of the reaction involving 4-chloroaniline decreased with lower amounts of NaBH_4_ (1 mmol) (Table 1, entry 4) and shorter reaction times (Table 1, entries 5, and 6). Other reducing agents such as hydrazine, formic acid and glycerol were investigated, giving lower yields of the expected amine (Table 1, entries 7–9). Using other solvents such as CH_3_CN, DMF and toluene obtained lower yields (Table 1, entries 10–12). Therefore, conditions including the catalyst with 0.15 mol% of Au, 0.35 mol% of Cu, NaBH_4_ as reducing agent, in neat H_2_O, at room temperature, were selected as the optimal reaction conditions. This reference reaction, in the absence of the catalyst, did not make any progress (Table 1, entry 13). To highlight the advantage of using bimetallic Fe_3_O_4_@Phos-IL-AuCu, we examined the activity of single-metal catalysts, Fe_3_O_4_@Phos-IL-Au and Fe_3_O_4_@Phos-IL-Cu, in this reaction and using the optimized conditions. The experimental results indicated that both single-metal catalysts offered poorer yields than the reaction performed with the bimetallic catalyst (Table 1, entries 14, and 15). The higher catalytic activity of Fe_3_O_4_@Phos-IL-AuCu, as these results indicate, is due to the synergistic effect between the Cu and Au species.

**Table 1:** Optimization of reaction conditions for reduction of 4-chloronitrobenzene.


Entry	Cat. Au (mol%)	Cat. Cu (mol%)	Solvent	t (h)	Reductant	Yield (%)
1	0.06	0.16	H_2_O	2	NaBH_4_	50
2	0.1	0.24	H_2_O	2	NaBH_4_	65
3	0.15	0.35	H_2_O	2	NaBH_4_	100
4	0.15	0.35	H_2_O	2	NaBH_4_	61^a^
5	0.15	0.35	H_2_O	0.5	NaBH_4_	10
6	0.15	0.35	H_2_O	1	NaBH_4_	40
7	0.15	0.35	H_2_O	2	Hydrazine	2
8	0.15	0.35	H_2_O	2	Glycerol	0
9	0.15	0.35	H_2_O	2	Formic acid	0
10	0.15	0.35	CH_3_CN	2	NaBH_4_	25
11	0.15	0.35	DMF	2	NaBH_4_	20
12	0.15	0.35	Toluene	2	NaBH_4_	0
13	–	–	H_2_O	2	NaBH_4_	0^b^
14	0.15	–	H_2_O	2	NaBH_4_	11^c^
15	–	0.35	H_2_O	2	NaBH_4_	7^d^

Reaction conditions: 4-chloro nitrobenzene (0.5 mmol), reductant agent (2 mmol), solvent (1.4 mL).

Yields determined by GC.

^a^Reaction performed in the presence of 1 mmol of NaBH_4_.

^b^Reaction performed in the absence of the catalyst.

^c^Reaction performed using 0.15 mol% Au.

^d^Reaction performed using 0.35 mol% Cu.

After determination of optimum reaction conditions, the reduction of different nitroarene compounds was assessed (Table 2). The results showed that high to excellent yields were obtained in the reduction of nitro aromatic compounds with both groups of electron‐donating groups such as –Me, –CH_2_OH, NH_2_, and –OH and electron‐withdrawing groups such as –F, –Cl, –Br, –CN, and –COMe. In general, the results show that the reduction of electron-rich nitroarenes was slower than the substrates containing electron‐deficient arrangements (Table 2, entries 2–15). Also, in the example run with 4-nitrobenzaldehyde, the aldehyde group was reduced along with the nitro group, (Table 2, entry 13). However, during the reduction of acetylnitroarenes only the nitro group was reduced (Table 2, entries 14, 15). In addition, nitro aromatic compounds with two nitro groups were completely reduced, using a larger amount of NaBH_4_ over longer times (Table 2, entries 16–19). Also, compounds with both electron‐donating and electron‐withdrawing substitutions had good yields (Table 2, entry 20). Reduction of 4-nitrobenzylchloride was performed very good and the corresponded amine obtained in excellent yield (Table 2, entry 21). Finally, the reduction of heterocyclic nitro compounds was carried out efficiently and the expected amines were isolated in 83–89% yields (Table 2, entries 22-24). Besides, the carbonyl group of the amide remained intact (Table 2, entries 22 and 23). Totally results of Table 2 showed achieving high TOF and TON of present bimetallic AuCu catalyst in reduction of nitroarenes.

**Table 2:**
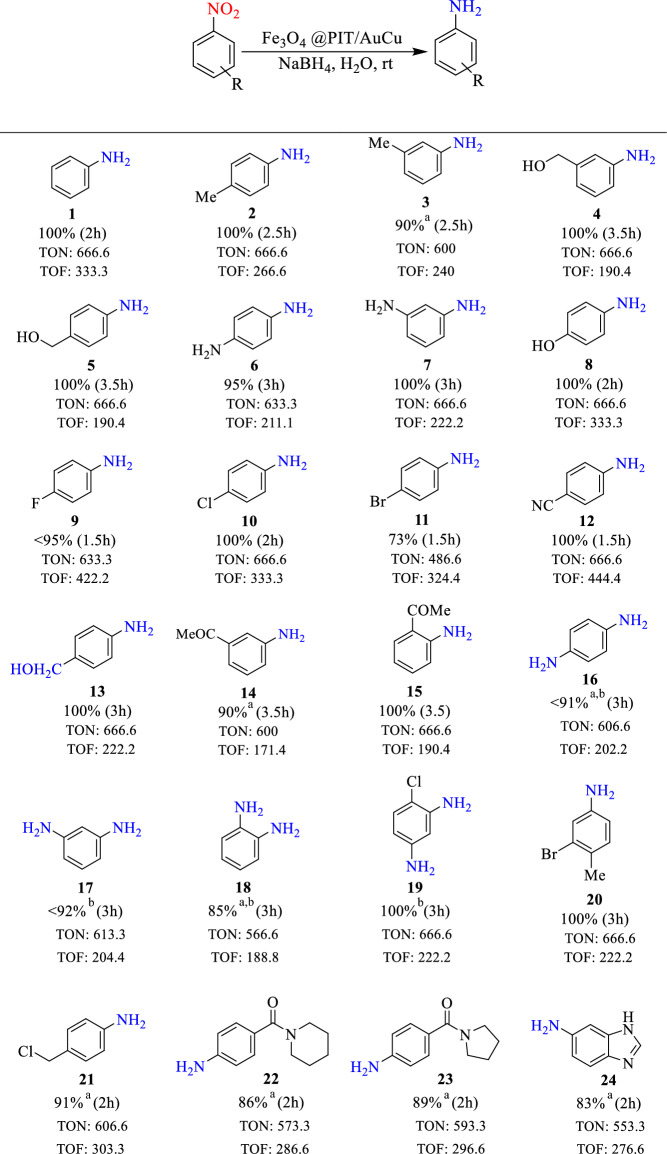
Reduction of assorted nitroarenes using Fe_3_O_4_@Phos-IL-AuCu as catalyst.

Nitro-compound (0.5 mmol), NaBH_4_ (2 mmol), catalyst (0.15 mol% Au, 0.35 mol% Cu, 25 mg), H_2_O (1.4 mL), rt.

Yield determined by GC.

TON (respect to Au)]; [TOF] (respect to Au) values [TON/time of reaction (h)].

^a^Isolated yield.

^b^NaBH_4_ (4 mmol).

**Figure 8:**
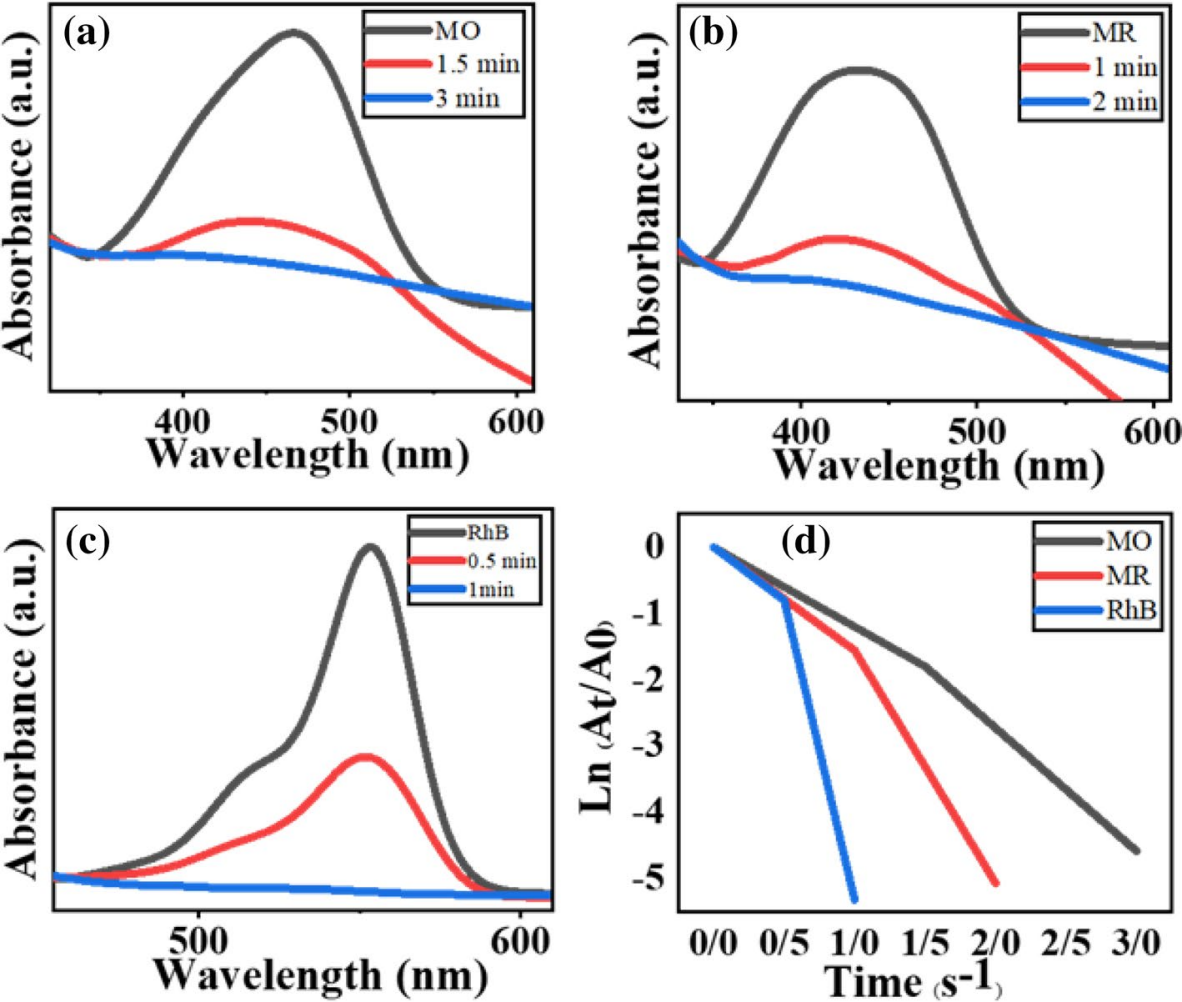
UV–Visible spectra for the reductive degradation of (**a**) MO, (**b**) MR, and (**c**) RhB in the presence of bimetallic Fe_3_O_4_@Phos-IL-AuCu catalyst and NaBH_4_ and (**d**) diagrams of ln (At/A0) versus reaction time for the reduction of listed dyes.

The catalytic performance of Fe_3_O_4_@Phos-IL-AuCu in the reductive degradation of organic dyes, including methyl red (MR), methyl orange (MO), and rhodamine B (RhB) was evaluated. The progresses of reactions were recorded by using UV–Vis spectrophotometer. The absorption of all MR (λmax = 435 nm), MO (λmax = 467 nm), and RhB (λmax = 556 nm) dyes decreased in time when using Fe_3_O_4_@Phos-IL-AuCu as catalyst together with NaBH_4_ as reducing agent. Generally, MO (λmax = 467 nm), MR (λmax = 435), and RhB (λmax = 556 nm) dyes completely declined in 3 min, 2 min, and 1 min, respectively, and during the desired times all dyes became colorless (Fig. 8a–c)^70,71^. Diagrams of ln (At/A0) vs. reaction time for the dyes reduction were drawn and the rate constants (k) of MO, MR, and RhB were 1.5, 2.1, and 2.6 min^−1^, respectively, which indicates good performance of the titled synthesized catalyst (Fig. 8d). TOF and TON for the reduction of dyes are calculated and shown in Table S2.

In order to compare the activity of the prepared bimetallic catalyst with the activity of the single metal catalysts such as Fe_3_O_4_@Phos-IL-Au and Fe_3_O_4_@Phos-IL-Cu, the reduction of dyes MO, MR, and RhB was investigated (Fig. S2). The results showed that 31.5%, 27%, and 11.5% yields were obtained in the reduction of MO, MR, and RhB dyes when using the Fe_3_O_4_@Phos-IL-Au catalyst. Also, 5%, 3%, and 9% yields were obtained in the reduction of MO, MR, and RhB dyes with the Fe_3_O_4_@Phos-IL-Cu catalyst. These results confirmed the higher catalytic activity of Fe_3_O_4_@Phos-IL-AuCu due to the synergetic effect between Cu and Au species.

The proposed mechanisms for the reduction of nitroarenes and organic dyes using the Fe_3_O_4_@Phos-IL-AuCu catalyst are based on the well-established principles of metal-catalyzed hydrogenation, as depicted in Figs. S6 and S7. The process begins with reaction of NaBH_4_ with H_2_O and generation of hydrogen and NaBO_2_. Produced hydrogen, along with the nitroarenes or organic dyes, are adsorbed onto the surface of the Fe_3_O_4_@Phos-IL-AuCu catalyst and performed the reduction of functional groups^72,73^.

**Figure 9:**
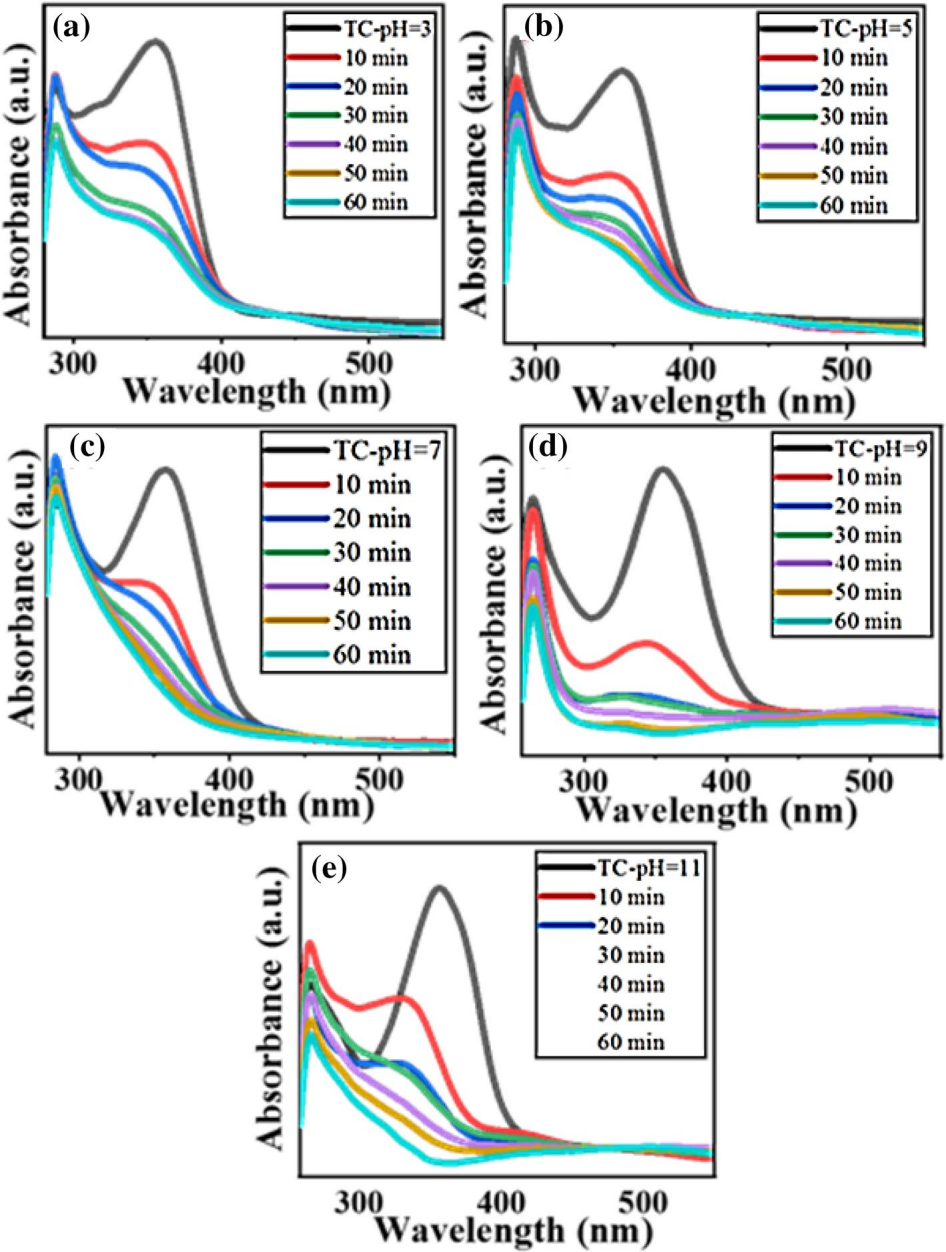
UV–Visible spectra for the degradation of tetracycline (TC) in the presence of Fe_3_O_4_@Phos-IL-AuCu and amoniumperoxodisolfates, in (**a**) pH: 3; (**b**) pH: 5; (**c**) pH: 7; (**d**) pH: 9; and (**e**) pH: 11.

Finally, the catalytic activity of the as-prepared Fe_3_O_4_@Phos-IL-AuCu catalyst has been assessed in the degradation of tetracycline (TC) by using ammonium peroxodisulfate at diverse pH levels (3, 5, 7, 9, and 11) (Fig. 9a–e). The reaction process was recorded using the UV–Vis spectrophotometer. Results showed that the absorbance of tetracycline gradually decreased with increasing reaction time, and, similarly to the experience in previous reports, the best result was obtained in alkaline media.

Degradation of tetracycline with Fe_3_O_4_@Phos-IL-Au (Fig. S3) and Fe_3_O_4_@Phos-IL-Cu (Fig. S4) was also investigated. Here, the higher efficiency of bimetallic catalyst Fe_3_O_4_@Phos-IL-AuCu is demonstrated once more versus the reactions run in the presence of monometallic catalytic species (Table S1).

**Scheme 2:**
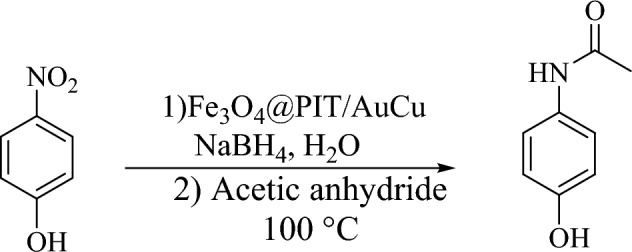
Preparation of acetaminophen via a one-pot reduction/amidation of 4-nitrophenol and acetic anhydride.

Since the reduction of 4-nitrophenol proceeded very efficiently (Table 2, entry 8), we studied the synthesis of acetaminophen via a one-pot reduction/amidation of 4-nitrophenol. Results show that the reaction proceeded very effectively and the desired product was obtained in 84% isolated yield (Scheme 2).

**Figure 10:**
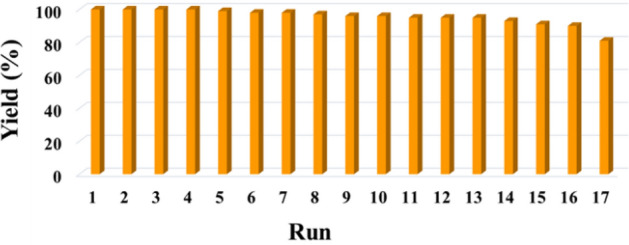
Recycling of the catalyst Fe_3_O_4_@Phos-IL-AuCu during the reduction of 4-nitrophenol.

Efficiently recovering and reusing magnetic heterogeneous catalysts is crucial for both environmental and economic perspectives. In this regard, the recovery and the recycling facilities of Fe_3_O_4_@Phos-IL-AuCu were studied in the reduction of 4-nitrophenol under optimized reaction conditions (Fig. 10). To achieve this objective, the catalyst was recovered after 30 min using an external magnet, washed with ethyl acetate, and after drying, it was utilized in another new batch of the reaction. According to these findings, the catalyst demonstrated the ability to be reused for 16 consecutive runs with only a slight decrease in activity. However, during the 17th run, the yield dropped to 81%.

**Figure 11:**
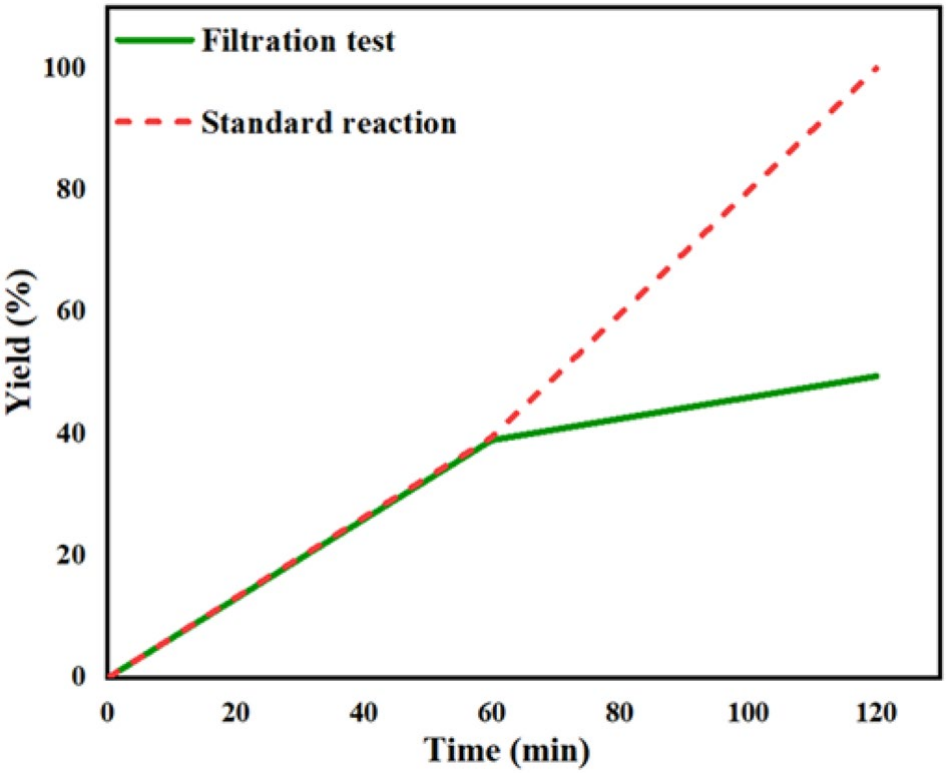
Filtration tests results for the catalyst Fe_3_O_4_@Phos-IL-AuCu during the reduction of 4-chloronitrobenzene.

To determine whether the Fe_3_O_4_@Phos-IL-AuCu is heterogeneous or homogeneous in nature, the filtration test was used for the reduction of 4-chloronitrobenzene under optimal conditions. During the course of the experiment, the reaction proceeded to a level of 39% completion after an initial reaction time of 1 h. At this stage, the reaction mixture was subjected to filtration, following which the filtrate was allowed to continue the reaction for an additional hour. Subsequent analysis using gas chromatography (GC) revealed that the progress of the reaction was slowed down and the product was formed at a yield of only 49% (Fig. 11). This result indicated negligible leaching of active catalysts into the reaction mixture and the possible heterogeneous nature of the catalyst.

**Figure 12:**
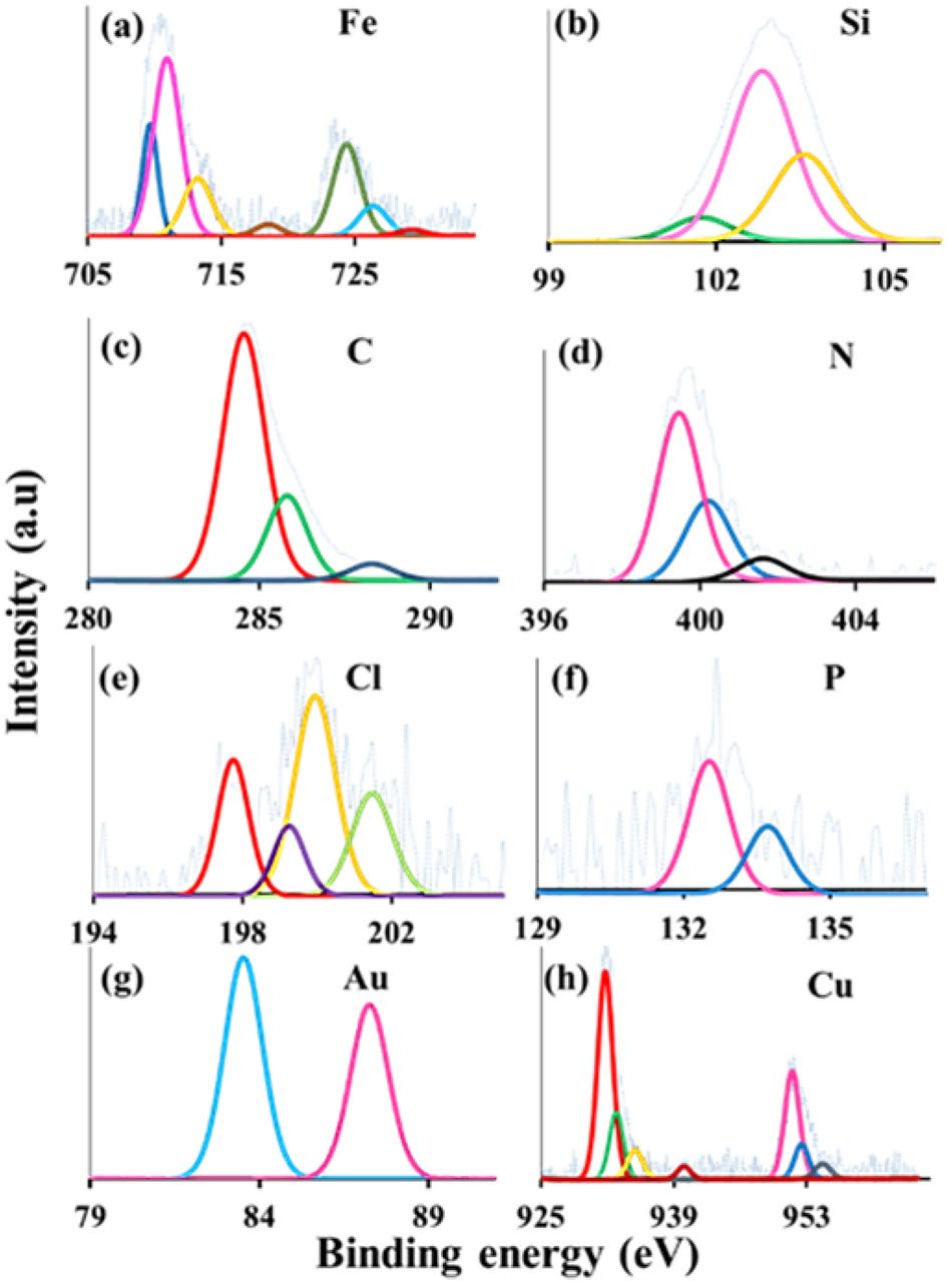
XPS spectrum of the reused catalyst after 17th run in Fe, Si, C, N, Cl, P, Au, and Cu regions.

**Figure 13:**
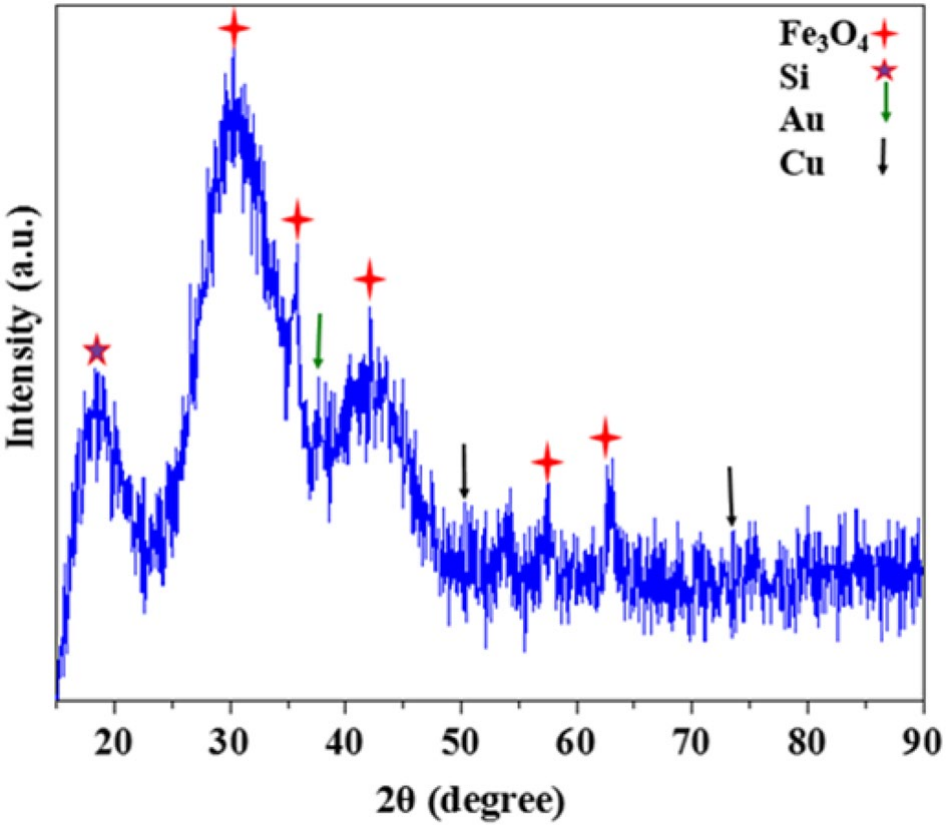
XRD pattern of the catalyst after 17th run.

**Figure 14:**
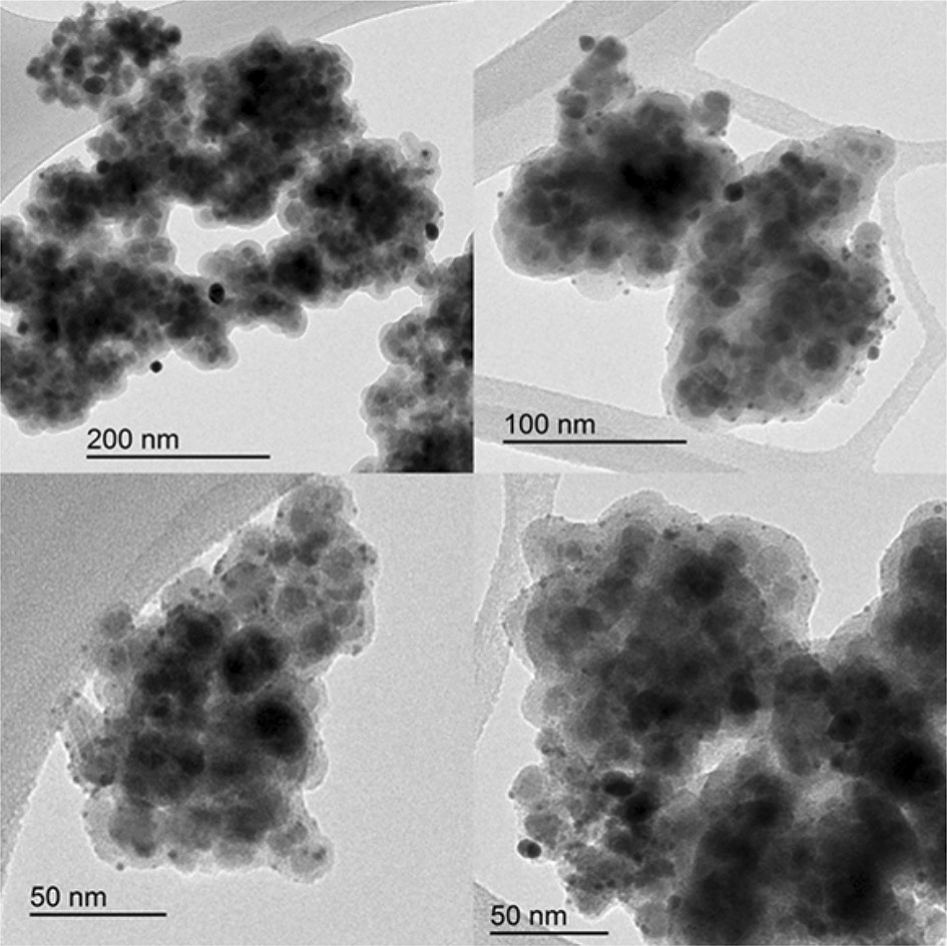
TEM images of the catalyst after 17th run.

In order to investigate the stability of the catalyst, the structure of the reused catalyst was analyzed by VSM (Fig. 7), XPS (Fig. 12), XRD (Fig. 13), and TEM (Fig. 14). VSM analysis of the reused catalyst showed the preservation of the magnetic property of the catalyst during recycling. The information revealed by the XPS spectrum in the Fe, Si, C, N, Cl, P, Au, and Cu regions indicated that similar patterns of elements to those of the fresh catalyst existed, confirming the high stability of the catalyst during the reaction.

Furthermore, the XRD spectra obtained from the reused catalyst showed peaks corresponding to Fe_3_O_4_, Si, Au, and Cu, which are close to the analogous ones detected in the newly prepared catalyst, providing evidence of the catalyst’s durability (Fig. 13).

TEM images of reused catalysts also showed a very similar pattern to that of fresh catalysts with small increase in average size of nanoparticles, confirming the stability of the catalyst (Fig. 14, Fig. S5).

## Experimental

### Materials

Copper(II) sulfate pentahydrate (CuSO_4_·5H_2_O), sodium tetrachloroaurate(III) dihydrate (NaAuCl_4_·2H_2_O), iron(III) chloride hexahydrate (FeCl_3_·6H_2_O), iron(II) chloride tetrahydrate (FeCl_2_·4H_2_O), silicon dioxide (SiO_2_), (3-glycidyloxypropyl)trimethoxysilane, polyvinylpyrrolidone (PVP, MW ~ 10,000), imidazole, propargyl bromide, chlorodiphenylphosphine, sodium borohydride (NaBH_4_), methyl orange (MO), sodium azide, 3-chloro-1,2-propanediol, ascorbic acid, rhodamine B (RhB), nitro compounds, methyl red (MR), and tetracycline (TC) were purchased from Sigma-Aldrich, Acros and Merck MilliporeSigma.

### Synthesis of Fe_3_O_4_ NPs

Firstly, FeCl_3_⋅6H_2_O (16 mmol, 4.3 g), FeCl_2_⋅4H_2_O (8 mmol, 1.6 g), and distilled water (70 mL) were mixed and stirred under an argon atmosphere at room temperature for 10 min. After that, aqueous ammonia (25%, 10 mL) was gradually poured, and the suspension stirred at 80 °C for 4 h. Finally, an external magnet allowed for the separation of the magnetic particles which were washed with distilled H_2_O (3 × 20 mL) and EtOH (3 × 20 mL), and dried in the oven at 60 °C.

### Synthesis of Fe_3_O_4_@SiO_2_

Fe_3_O_4_ nanoparticles (2 g) were sonicated in ethanol (200 mL) for 30 min, and tetraethyl orthosilicate (3 mL) and aqueous ammonia (25 %, 12 mL) were added under the argon atmosphere, respectively. The resultant mixture was stirred at room temperature for 24 h. Afterwards, Fe_3_O_4_@SiO_2_ nanoparticles were separated using an external magnet, washed with distilled water (3 × 20 mL) and ethanol (3 × 20 mL), and dried overnight in the oven at 60 °C.

### Synthesis of Fe_3_O_4_@SiO_2_@epoxide (I)

Freshly obtained Fe_3_O_4_@SiO_2_ (1 g) was suspended in dry toluene (30 mL) and then sonicated for 30 min. Next, (3-glycidyloxypropyl)trimethoxysilane (5 mmol, 1.1 mL) was added to the above mixture under argon atmosphere. The suspension was stirred at 105 °C for 1 day. The Fe_3_O_4_@SiO_2_@epoxide product was separated magnetically, washed repeatedly with distilled water and dichloromethane, and lastly dried at 60 °C for 4 h.

### Synthesis of 1-(prop-2-yn-1-yl)-1H-imidazole (II)

Sodium hydride (60%, 9 mmol, 360 mg) was added to a 25 mL flask under argon protection. Then, dry THF (6 mL) was added and flask was placed in an ice bath. Next, crystallized imidazole (9 mmol, 612 mg) was dissolved in 2 mL of dry THF and added to the mixture. After 5 min, propargyl bromide (7.5 mmol, 0.56 mL) diluted in 1 mL THF was added and mixture was stirred at room temperature for 24 h. Afterward, solvent was removed using a rotary evaporator and water (10 mL) and CH_2_Cl_2_ (10 mL) were added to the residual mixture. The organic phase containing the product was separated and its solvent was evaporated. This washing process with water was repeated three additional times. Finally, an amber-colored product (II) was obtained.

### Synthesis of Fe_3_O_4_@C_2_H_4_OPPh_2_@1-(2-propynyl)-1H-imidazole

Fe_3_O_4_@SiO_2_@epoxide (1 g) and 1-(2-propynyl)-1H-imidazole (5 mmol, 0.53 g) were sonicated in dry dichloromethane for 15 min, and next, chlorodiphenylphosphine (5 mmol, 0.9 mL) was added to the mixture and stirred for 24 h at 50 °C. Lastly, an external magnet was used to separate the solid, which was washed with distilled water (2 × 20 mL) and dichloromethane (2 × 20 mL), and dried in the oven at 60 °C overnight.

### Synthesis of Fe_3_O_4_@Phos-IL

Sodium azide (8 mmol, 0.52 g) and 3-Chloro-1,2-propanediol (5 mmol, 0.4 mL) were dissolved in *N,N*-dimethylformamide (10 mL), and then the reaction mixture was stirred at 100 °C for 24 h. In the next step, the mixture was cooled to room temperature and the phosphine functionalized magnetic NPs (1 g) were added to the mixture. Then, a solution containing copper(II) sulfate (0.09 mmol, 14.3 mg) and ascorbic acid (0.17 mmol, 30 mg) was mixed in DMF (1 mL) and the final solution was stirred at 70 °C for 24 h. The precipitate was separated employing an external magnet and washed repeatedly with distilled water (20 mL) and ethyl acetate (20 mL) and dried at 70 °C for 24 h.

### Synthesis of Fe_3_O_4_@Phos-IL-AuCu

A suspension of Fe_3_O_4_@Phos-IL (300 mg) in distilled water (6 mL) was sonicated for 15 min, and then a solution of CuSO_4_∙5H_2_O (7.5 mg) and NaAuCl_4_·2H_2_O (7.5 mg) in H_2_O (2 mL) was added to the mixture and then stirred for 20 min in an ice bath under the argon atmosphere. Next, NaBH_4_ (0.6 mmol, 22.6 mg) in H_2_O (1 mL) was added slowly and the reaction stirred for 1 day at 25 °C. Finally, the resulting solid was separated and treated as it was described above.

### Catalytic reduction of nitro compounds

Nitroarene (0.5 mmol), catalyst (25 mg containing 0.15 mol% Au and 0.35 mol% Cu), NaBH_4_ (2 mmol, 75 mg), were mixed in H_2_O (1.4 mL), and then the mixture was stirred at room temperature. The reaction was monitored by GC analysis and stopped when it was judged complete. The crude substance was treated with ethyl acetate (3 × 5 mL), and the organic residue was purified by chromatography (column or plate).

### General procedure for the catalytic reduction of MO, MR, and RhB dyes

2.5 mL of the desired dye’s solution (MO, MR, or RhB, 0.06 mM), the catalyst (1 mg) and 0.5 mL of an aqueous solution of NaBH_4_ (0.5 M) were poured into a quartz cuvette, and the mixture was stirred at room temperature. UV–Visible spectroscopy was selected to monitor the progress of the reaction.

### Typical procedures for the degradation of TC

TC (0.005 mmol, 2.5 mg), catalyst (15 mg), and H_2_O (50 mL) were poured into an Erlenmeyer flask and stirred for 3 min, and then ammonium peroxydisulfate (1 mmol, 240 mg) was added to the mixture. Again, the course of the reaction was monitored every 10 min through UV–Visible spectroscopy.

### One-pot synthesis of acetaminophen

In a 5 mL flask, 4-nitrophenol (0.4 mmol, 55.6 mg), NaBH_4_ (1.6 mmol, 60.5 mg), catalyst (20 mg), and H_2_O (1.5 mL) were added and the reaction was stirred at 25 °C. After 2 h, acetic anhydride (1 mmol, 0.1 mL) was poured into the flask, and the reaction mixture was stirred for an additional 2 hours at 100 °C. The crude material was extracted with ethyl acetate (3×5 mL), purified by column/plate chromatography, and acetaminophen was obtained in 84% isolated yield. It was characterized only by ^1^H NMR.

## Conclusion

In conclusion, a new magnetic and recyclable bimetallic Fe_3_O_4_@Phos-IL-AuCu catalyst was synthesized. This catalyst showed high activity in reducing nitro compounds, reduction of organic dyes, and degrading tetracycline. It also promoted all three of these reactions at room temperature in water as a green solvent. The Fe_3_O_4_@Phos-IL-AuCu catalyst showed the highest activity in comparison with its single metal species such as Fe_3_O_4_@Phos-IL-Au, and Fe_3_O_4_@Phos-IL-Cu in all three reactions mentioned, which confirms the synergistic effects between gold and copper particles. In addition, the synthesized catalyst can be easily recycled due to its magnetic properties and reused up to 17 times without noticeable decrease in its activity.

### Supplementary Information

Supplementary Information.

## Data Availability

All data generated or analyzed during this study are included in this published article.

## References

[1] Gholinejad M, Zareh F, Sheibani H, Nájera C, Yus M (2022). Magnetic ionic liquids as catalysts in organic reactions. J. Mol. Liq..

[2] Taheri-Ledari R, Rahimi J, Maleki A, Shalan AE (2020). Ultrasound-assisted diversion of nitrobenzene derivatives to their aniline equivalents through a heterogeneous magnetic Ag/Fe3O4-IT nanocomposite catalyst. New J. Chem..

[3] Rahimi J, Taheri-Ledari R, Niksefat M, Maleki A (2020). Enhanced reduction of nitrobenzene derivatives: Effective strategy executed by Fe3O4/PVA-10% Ag as a versatile hybrid nanocatalyst. Catal. Commun..

[4] Taheri-Ledari R, Mirmohammadi SS, Valadi K, Maleki A, Shalan AE (2020). Convenient conversion of hazardous nitrobenzene derivatives to aniline analogues by Ag nanoparticles, stabilized on a naturally magnetic pumice/chitosan substrate. RSC Adv..

[5] Hajizadeh Z, Valadi K, Taheri-Ledari R, Maleki A (2020). Convenient Cr(VI) removal from aqueous samples: Executed by a promising clay-based catalytic system, magnetized by Fe3O4 nanoparticles and functionalized with humic acid. ChemistrySelect.

[6] Khaleghi N, Forouzandeh-Malati M, Ganjali F, Rashvandi Z, Zarei-Shokat S, Taheri-Ledari R, Maleki A (2023). Silver-assisted reduction of nitroarenes by an Ag-embedded curcumin/melamine-functionalized magnetic nanocatalyst. Sci. Rep..

[7] Lei Z, Chen B, Koo YM, MacFarlane D (2017). R. Introduction: Ionic liquids. Chem. Rev..

[8] Vekariya RL (2017). A review of ionic liquids: Applications towards catalytic organic transformations. J. Mol. Liq..

[9] Gholinejad M, Razeghi M, Ghaderi A, Biji P (2016). Palladium supported on phosphinite functionalized Fe3 O4 nanoparticles as a new magnetically separable catalyst for Suzuki–Miyaura coupling reactions in aqueous media. Catal. Sci. Technol..

[10] Dorel R, Echavarren AM (2015). Gold (I)-catalyzed activation of alkynes for the construction of molecular complexity. Chem. Rev..

[11] Sugimoto K, Matsuya Y (2017). Recent applications of gold-catalyzed cascade reactions in total synthesis of natural product. Tetrahedron Lett..

[12] Pflästerer D, Hashmi ASK (2016). Gold catalysis in total synthesis—Recent achievements. Chem. Soc. Rev..

[13] Patil NT, Ambegave SB, More TR, Shubham NA (2023). Gold-based enantioselective bimetallic catalysis. Chem. Commun..

[14] Wang A, Liu XY, Mou CY, Zhang T (2013). Understanding the synergistic effects of gold bimetallic catalysts. J. Catal..

[15] Singh AK, Xu Q (2013). Synergistic catalysis over bimetallic alloy nanoparticles. ChemCatChem..

[16] Ellert OGG, Tsodikov MV, Nikolaev SA, Novotortsev VM (2014). Bimetallic nanoalloys in heterogeneous catalysis of industrially important reactions: Synergistic effects and structural organization of active components. Russ. Chem. Rev..

[17] Gholinejad M, Khosravi F, José MS, Vishnuraj R, Pullithadathil B (2023). Bimetallic AuNi nanoparticles supported on mesoporous MgO as catalyst for Sonogashira–Hagihara cross-coupling reaction. J. Organomet. Chem..

[18] Gholinejad M, Khezri R, Nayeri S, Vishnuraj R, Pullithadathil B (2022). Gold nanoparticles supported on NiO and CuO: The synergistic effect toward enhanced reduction of nitroarenes and A3-coupling reaction. Mol. Catal..

[19] Gholinejad M, Mozafari S, Nikfarjam N, Nayeri S, Sansano JM (2023). Bimetallic AuCo supported on magnetic crosslinked copoly (ionic liquid) nanohydrogel and study of its catalytic activity. Appl. Organomet. Chem..

[20] Han S, Mullins CB (2020). Catalytic reactions on Pd–Au bimetallic model catalysts. Acc. Chem. Res..

[21] Gholinejad M, Ahmadi J, Nájera C (2016). Silica microparticles supported gold and copper ferrite nanoparticles: A magnetically recyclable bimetallic catalyst for Sonogashira reaction. ChemistrySelect.

[22] Quiton KGN, Lu MC, Huang YH (2021). Synthesis and catalytic utilization of bimetallic systems for wastewater remediation: A review. Chemosphere.

[23] Nasrollahzadeh M, Sajjadi M, Maham M, Sajadi SM, Barzinjy AA (2018). Biosynthesis of the palladium/sodium borosilicate nanocomposite using *Euphorbia milii* extract and evaluation of its catalytic activity in the reduction of chromium (VI), nitro compounds and organic dyes. Mater. Res. Bull..

[24] Arumugam V, Moodley KG, Dass A, Gengan RM, Ali D, Alarifi S (2021). Ionic liquid covered iron-oxide magnetic nanoparticles decorated zeolite nanocomposite for excellent catalytic reduction and degradation of environmental toxic organic pollutants and dyes. J. Mol. Liq..

[25] Uddin MJ, Ampiaw RE, Lee W (2021). Adsorptive removal of dyes from wastewater using a metal–organic framework: A review. Chemosphere.

[26] Amangelsin Y, Semenova Y, Dadar M, Aljofan M, Bjørklund G (2023). The impact of tetracycline pollution on the aquatic environment and removal strategies. Antibiotics.

[27] Daghrir R, Drogui P (2013). Tetracycline antibiotics in the environment: A review. Environ. Chem. Lett..

[28] Saadati F, Keramati N, Ghazi MM (2016). Influence of parameters on the photocatalytic degradation of tetracycline in wastewater: A review. Crit. Rev. Environ. Sci. Technol..

[29] Gopal G, Alex SA, Chandrasekaran N, Mukherjee A (2020). A review on tetracycline removal from aqueous systems by advanced treatment techniques. RSC Adv..

[30] Singh G, Rani S, Arora A, Duggal H, Mehta D (2017). Organic–inorganic nano-hybrid decorated by copper (II) incarceration: A versatile catalytic assembly for the swift reduction of aromatic nitro and dye compounds. Mol. Catal..

[31] Goksu H, Sert H, Kilbas B, Sen F (2017). Recent advances in the reduction of nitro compounds by heterogenous catalysts. Curr. Org. Chem..

[32] Pansambal S, Roy A, Mohamed HEA, Oza R, Vu CM, Marzban A, Murthy HA (2022). Recent developments on magnetically separable ferrite-based nanomaterials for removal of environmental pollutants. J. Nanomater..

[33] Xu S, Li J, Chen L (2011). Molecularly imprinted polymers by reversible addition–fragmentation chain transfer precipitation polymerization for preconcentration of atrazine in food matrices. Talanta.

[34] Lien YH, Wu TM, Wu JH, Liao JW (2011). Cytotoxicity and drug release behavior of PNIPAM grafted on silica-coated iron oxide nanoparticles. J. Nanopart. Res..

[35] Kizil R, Irudayaraj J, Seetharaman K (2002). Characterization of irradiated starches by using FT-Raman and FTIR spectroscopy. J. Agric. Food Chem..

[36] Domínguez-Crespo MA (2020). New triazole and isoxazole compounds as corrosion inhibitors for Cu–Ni (90/10) alloy and galvanized steel substrates. Metall. Mater. Trans..

[37] Li K, Qian L, Song W, Zhu M, Zhao Y, Miao Z (2018). Preparation of an ionic liquid-based hydrogel with hyperbranched topology for efficient removal of Cr (VI). J. Mater. Sci..

[38] Ahmadi L, Ahmadi E, Mohamadnia Z (2021). Imidazolium-based poly (ionic liquid)s for demulsification of water in crude oil emulsions. Polym. Adv. Technol..

[39] Firouzabadi H, Iranpoor N, Ghaderi A, Gholinejad M, Rahimi S, Jokar S (2014). Design and synthesis of a new phosphinite-functionalized clay composite for the stabilization of palladium nanoparticles. Application as a recoverable catalyst for C–C bond formation reactions. RSC Adv..

[40] Wang J, Liu F (2013). Hydrophilic simultaneous interpenetrating polymer network silicone hydrogels prepared by hybrid photopolymerization. Des. Monomers Polym..

[41] Li H, Zheng Q, Han C (2010). Click synthesis of podand triazole-linked gold nanoparticles as highly selective and sensitive colorimetric probes for lead (II) ions. Analyst.

[42] Nayak S, Mohapatra L, Parida K (2015). Supporting information visible light driven novel gC 3N4/NiFe-LDH composites photocatalyst with enhanced photocatalytic activity towards water oxidation and reduction reaction. J. Mater. Chem. A.

[43] Osuga R, Yokoi T, Kondo JN (2019). IR observation of activated ether species on acidic OH groups on H-ZSM-5 zeolites. Mol. Catal..

[44] Pervez MN, He W, Zarra T, Naddeo V, Zhao Y (2020). New sustainable approach for the production of Fe3O4/graphene oxide-activated persulfate system for dye removal in real wastewater. Water.

[45] Arrigo R, Schuster ME (2019). On the high structural heterogeneity of Fe-impregnated graphitic-carbon catalysts from Fe nitrate precursor. Catalysts.

[46] Gholinejad M, Afrasi M, Najera C (2019). Caffeine gold complex supported on magnetic nanoparticles as a green and high turnover frequency catalyst for room temperature A3 coupling reaction in water. Appl. Organomet. Chem..

[47] Díaz EJC, Gálvez-Martínez S, Vico MCT, González MPV, Mateo-Martí E (2020). 2-D organization of silica nanoparticles on gold surfaces: CO2 marker detection and storage. RSC Adv..

[48] Meškinis Š, Vasiliauskas A, Andrulevičius M, Peckus D, Tamulevičius S, Viskontas K (2020). Diamond like carbon films containing Si: Structure and nonlinear optical properties. Materials.

[49] Xia K, Liu X, Liu H, Lu Y, Liu Z, Li Y, Wang D (2021). Carbon-enriched SiOC ceramics with hierarchical porous structure as anodes for lithium storage. Electrochim. Acta.

[50] Li B, Lai C, Zeng G, Qin L, Yi H, Huang D, Liu S (2018). Facile hydrothermal synthesis of Z-scheme Bi2Fe4O9/Bi2WO6 heterojunction photocatalyst with enhanced visible light photocatalytic activity. ACS Appl. Mater. Interfaces.

[51] Yuan S, Gu J, Zheng Y, Jiang W, Liang B, Pehkonen SO (2015). Purification of phenol-contaminated water by adsorption with quaternized poly (dimethylaminopropyl methacrylamide)-grafted PVBC microspheres. J. Mater. Chem..

[52] Gholinejad M, Mirmohammadi S, Sansano JM (2021). Novel water dispersible and magnetically recoverable palladium nano catalyst for room-temperature Suzuki–Miyaura coupling reaction. ChemistrySelect.

[53] Ech-Chamikh E, Essafti A, Ijdiyaou Y, Azizan M (2006). XPS study of amorphous carbon nitride (aC: N) thin films deposited by reactive RF sputtering. Sol. Energy Mater. Sol. Cells.

[54] Félix R, Llobera-Vila N, Hartmann C, Klimm C, Hartig M, Wilks RG, Bär M (2018). Preparation and in-system study of SnCl2 precursor layers: Towards vacuum-based synthesis of Pb-free perovskites. RSC Adv..

[55] Xie FX, Zhang D, Su H, Ren X, Wong KS, Grätzel M, Choy WC (2015). Vacuum-assisted thermal annealing of CH3NH3PbI3 for highly stable and efficient perovskite solar cells. ACS Nano.

[56] Morgan DJ (2015). Resolving ruthenium: XPS studies of common ruthenium materials. Surf. Interface Anal..

[57] Strydom CA, Van Staden JF, Strydom HJ (1990). An XPS investigation of silver chloride coated ion-selective electrodes. J. Electroanal. Chem. Interfacial Electrochem..

[58] Wang Y, Lan Z, Xu F, Zhao L, Xu J, Chen J, Ma Z (2022). Exploration of phosphorus oxide in heavily phosphorus-doped polysilicon films of tunneling oxide passivation contact solar cells. ACS Appl. Electron. Mater..

[59] Sun J, Zheng G, Lee HW, Liu N, Wang H, Yao H, Cui Y (2014). Formation of stable phosphorus–carbon bond for enhanced performance in black phosphorus nanoparticle–graphite composite battery anodes. Nano Lett..

[60] Gholinejad M, Dasvarz N, Shojafar M, Sansano JM (2019). Starch functionalized creatine for stabilization of gold nanoparticles: Efficient heterogeneous catalyst for the reduction of nitroarenes. Inorgan. Chim. Acta.

[61] Wang W, Mai Z, Chen Y, Wang J, Li L, Su Q, Hong X (2017). A label-free fiber optic SPR biosensor for specific detection of C-reactive protein. Sci. Rep..

[62] Xu L, Yang Y, Hu ZW, Yu SH (2016). Comparison study on the stability of copper nanowires and their oxidation kinetics in gas and liquid. ACS Nano.

[63] Tran PD, Nguyen M, Pramana SS, Bhattacharjee A, Chiam SY, Fize J, Barber J (2012). Copper molybdenum sulfide: A new efficient electrocatalyst for hydrogen production from water. Energy Environ. Sci..

[64] Nurdan KY, Fatma KB, Koç MM, Dilek N (2020). Characterization of magnetic Fe3 O4@ SiO2 nanoparticles with fluorescent properties for potential multipurpose imaging and theranostic applications. J. Mater. Sci. Mater. Electron..

[65] Dagher S, Soliman A, Ziout A, Tit N, Hilal-Alnaqbi A, Khashan S, Qudeiri JA (2018). Photocatalytic removal of methylene blue using titania- and silica-coated magnetic nanoparticles. Mater. Res. Express.

[66] Corona JE, Maldonado RD, Oliva AI (2007). Vacuum oven to control the annealing process in alloyed nanolayers. Rev. Mex. Fis..

[67] Volker E, Williams FJ, Calvo EJ, Jacob T, Schiffrin DJ (2012). O2 induced Cu surface segregation in Au–Cu alloys studied by angle resolved XPS and DFT modelling. Phys. Chem. Chem..

[68] Latif-ur-Rahman A, Shah A, Han C, Jan AK (2020). Monitoring of anthracene using nanoscale Au–Cu bimetallic alloy nanoparticles synthesized with various compositions. ACS Omega.

[69] Liu X, Wang A, Zhang T, Su DS, Mou CY (2011). Au–Cu alloy nanoparticles supported on silica gel as catalyst for CO oxidation: Effects of Au/Cu ratios. Catal. Today.

[70] Budi CS, Deka JR, Hsu WC, Saikia D, Chen KT, Kao HM, Yang YC (2021). Bimetallic Co/Zn zeolitic imidazolate framework ZIF-67 supported Cu nanoparticles: An excellent catalyst for reduction of synthetic dyes and nitroarenes. J. Hazard. Mater..

[71] Xi J, Wang Q, Duan X, Zhang N, Yu J, Sun H, Wang S (2021). Continuous flow reduction of organic dyes over Pd–Fe alloy based fibrous catalyst in a fixed-bed system. Chem. Eng. Sci..

[72] Nagshbandi Z, Gholinejad M, Sansano JM (2023). Novel magnetic zeolitic imidazolate framework for room temperature enhanced catalysis. Inorg. Chem. Commun..

[73] Gholinejad M, Iranpanah M, Karimi S, Sansano JM (2024). Cysteine and ionic liquid modified magnetic nanoparticles supported RuCu as a new bimetallic catalyst in reduction reactions. J. Mol. Struct..

