# Supplementary material for: Novel magnetic bimetallic AuCu catalyst for reduction of nitroarenes and degradation of organic dyes
Source: Sci Rep. 2024 Mar 11;14:5852. doi: 10.1038/s41598-024-56559-4 (PMC10925594; doi:10.1038/s41598-024-56559-4)
Supplement: Supplementary file 1 — Supplementary Information. [file 41598_2024_56559_MOESM1_ESM.docx]

**Novel Magnetic Bimetallic AuCu Catalyst for Reduction of Nitroarenes and Degradation of Organic Dyes**

Mohammad Gholinejad*^a,b^, Saba Bashirimousavi^a,^ José, M. Sansano^c^

a Department of Chemistry, Institute for Advanced Studies in Basic Sciences (IASBS), P. O. Box 45195‐1159, Gavazang, Zanjan 45137‐66731, Iran.

b Research Center for Basic Sciences & Modern Technologies (RBST), Institute for Advanced Studies in Basic Sciences (IASBS), Zanjan 45137‐66731, Iran.

c Departamento de Química Orgánica, Instituto de Síntesis Orgánica, and Centro de Innovación en Química Avanzada (ORFEO-CINQA), Universidad de Alicante, 03690-Alicante, Spain.

* Corresponding author: Mohammad Gholinejad, Email: gholinejad@iasbs.ac.ir

**
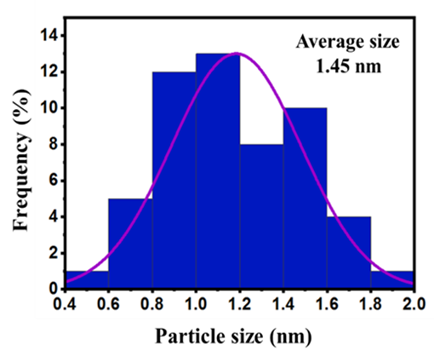
**

**Figure S1.** Particle size distribution diagram for Fe_3_O_4_@Phos-IL-AuCu


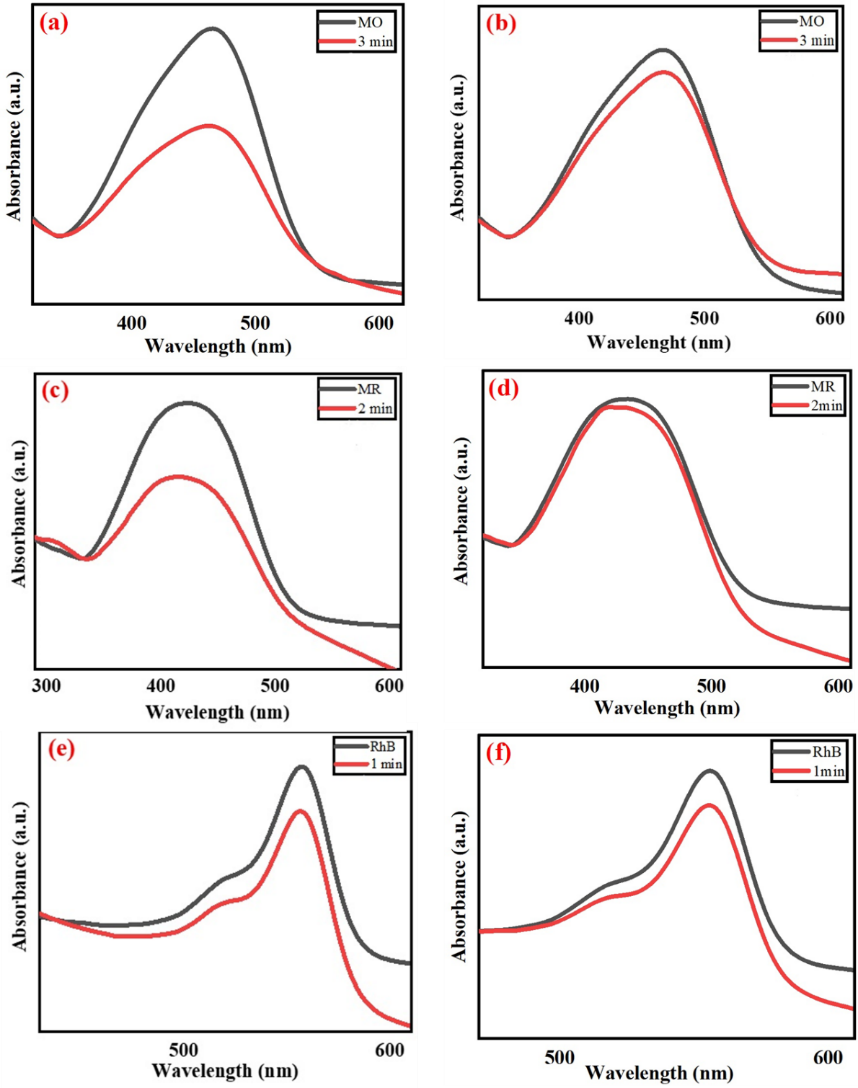


**Figure S2.** UV–visible spectra for the reductive degradation of MO, MR, and RhB in the presence of (a, c, e) Fe_3_O_4_@Phos-IL-Au; (b, d, f) Fe_3_O_4_@Phos-IL-Cu in the presence of NaBH_4._


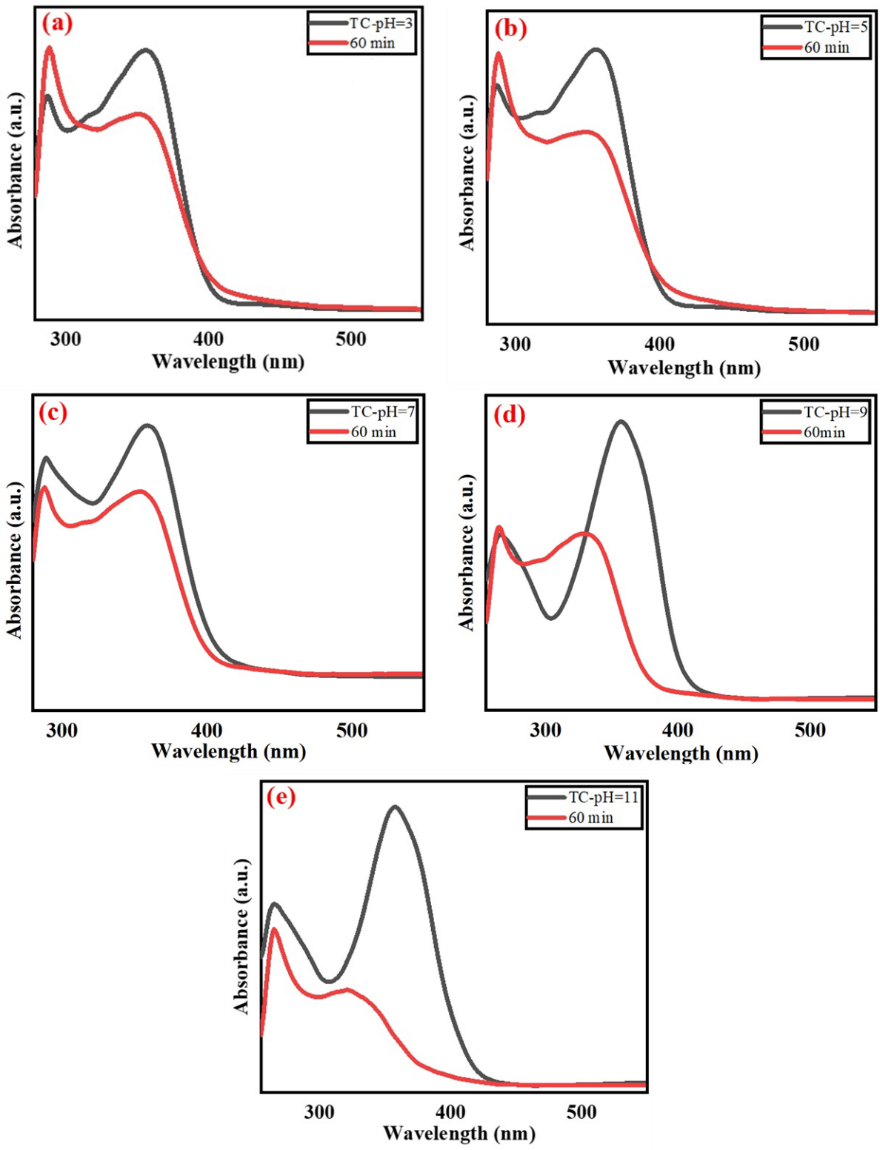


**Figure S3.** UV–visible spectra for the degradation of tetracycline (TC) in the presence of Fe_3_O_4_@Phos-IL-Au and amoniumperoxodisolfates, in (a) pH: 3, (b) pH: 5 (c) pH: 7 (d) pH: 9 and (e) pH: 11.


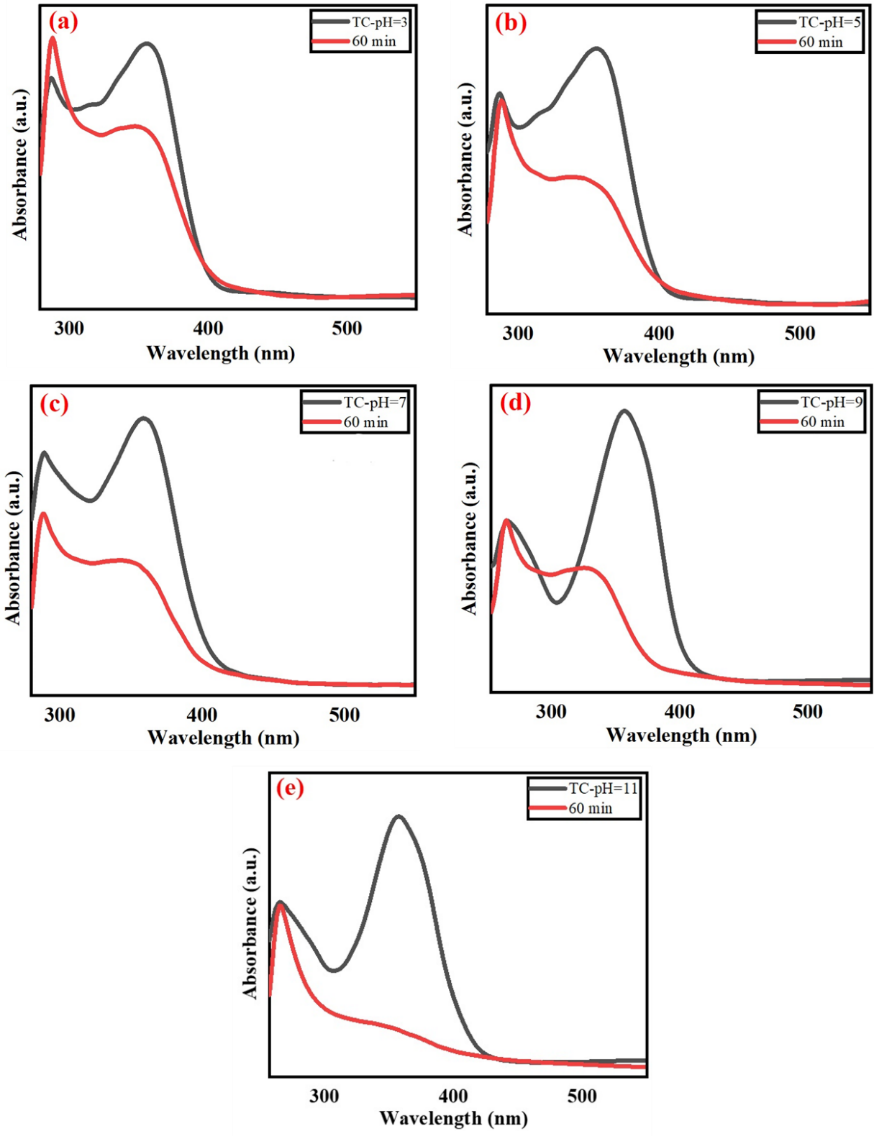


**Figure S4.** UV–visible spectra for the degradation of tetracycline (TC) in the presence of Fe_3_O_4_@Phos-IL-Cu and amoniumperoxodisolfates, in (a) pH: 3 (b) pH: 5 (c) pH: 7 (d) pH: 9 and (e) pH: 11.

**Table S1.** Degradation of tetracycline (TC) with Fe_3_O_4_@Phos-IL-AuCu, Fe_3_O_4_@Phos-IL-Au, and Fe_3_O_4_@Phos-IL-Cu in various pHs ^a, b^

|  | **Cat** | **pH:3** | **pH:5** | **pH:7** | **pH:9** | **pH:11** |
| --- | --- | --- | --- | --- | --- | --- |
| **Yield%** | Fe_3_O_4_@PIT/Au-Cu | 63% | 79% | 100% | 100% | 100% |
|  | Fe_3_O_4_@PIT/Au | 20% | 21% | 26% | 67% | 87% |
|  | Fe_3_O_4_@PIT/Cu | 30% | 51% | 53% | 80% | 91% |

[a] Reaction conditions: 50 ml of TC solution (0.005 w/w%), 240 mg amoniumperoxodisolfates, catalyst (15 mg), rt, 1h.

[b]Yield determined by UV–Visible spectroscopy.


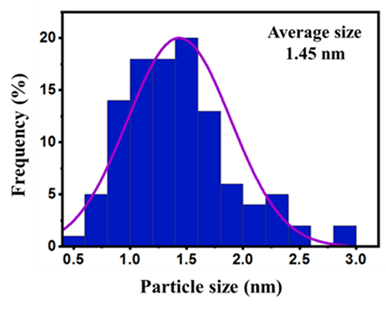


**Figure S5.** Particle size distribution diagram for reused Fe_3_O_4_@Phos-IL-AuCu after 17^th^ run.

**Table S2.** TON and TOF of the Fe_3_O_4_@Phos-IL-AuCu nanocatalyst in the reduction of organic dyes ^a, b^

| Entry | Organic dyes | Product | Time (min) | Yield (%)^b^ | TON^e^ | TOF^f^ |
| --- | --- | --- | --- | --- | --- | --- |
| 1 |  |  | 3 | 100 | 5 | 1.6 |
| 2 |  |  | 2 | 100 | 5 | 2.5 |
| 3 |  |  | 1 | 100 | 5 | 5 |
|  |  |  |  |  |  |  |

[a] Catalyst (1 mg).

[b] TON (respect to Au)]; [TOF] (respect to Au) values [TON/time of reaction (min)]

**Figure S6.** The proposed mechanism for the Fe_3_O_4_@Phos-IL-AuCu catalyzed nitroarenes reduction.^6,7^

**Figure S7.** The possible mechanism pathways for the reduction of organic dyes in Fe_3_O_4_@Phos-IL-AuCu catalytic system.^7^

**Characterization data of products**

**5-Aminobenzimidazole ^[1]^**

^1^H NMR (400 MHz, DMSO-*d*_6_) δ (ppm): 11.87 (s, 1H), 7.89 (s, 1H), 7.28 (d, *J*=8.5, 1H), 6.69 (d, *J*=2.0, 1H), 6.54 (dd, *J*=8.5, 2.0, 1H), 4.93 (s, 2H).

**(4-Aminophenyl)(piperidin-1-yl) methanone ^[2]^**

^1^H NMR (400 MHz, DMSO- *d_6_*) δ (ppm): 7.12 – 7.08 (m, 2H), 6.58 – 6.53 (m, 2H), 5.48 (s, 2H), 3.45 (t, *J*=5.4, 4H), 1.65 – 1.57 (m, 2H), 1.53 – 1.45 (m, 4H).

**(4-Aminophenyl)(pyrrolidin-1-yl)methanone ^[2]^**

^1^H NMR (400 MHz, DMSO-*d_6_*) δ = 7.29 (dd, _J_=8.5, 2.5, 2H), 6.55 (dd, _J_=8.6, 2.5, 2H), 5.53 (s, 2H), 3.45 (s, 4H), 1.82 (s, 4H).

**4-(Chloromethyl)aniline ^[3]^**

^1^H NMR (400 MHz, DMSO-*d_6_*) δ (ppm): 6.88 – 6.82 (m, 2H), 6.50 – 6.45 (m, 2H), 4.82 (s, 2H), 2.60 (s, 2H).

**3-Bromo-4-methylaniline** **^[4]^**

^1^H NMR (400 MHz, CDCl_3_-*d*) δ (ppm): 7.05 – 7.01 (m, 1H), 6.94 (d, *J*=2.4, 1H), 6.58 (dd, *J*=8.1, 2.4, 1H), 3.60 (s, 2H), 2.32 (s, 3H).

**Acetaminophen ^[5]^**

1 H NMR (400 MHz, DMSO) δ 9.68 (s, 1H), 9.17 (s, 1H), 7.37 (d, J = 8.9 Hz, 2H), 6.71 (d, J = 8.9 Hz, 2H), 2.01 (s,3H).

**^1^H NMR spectra:**


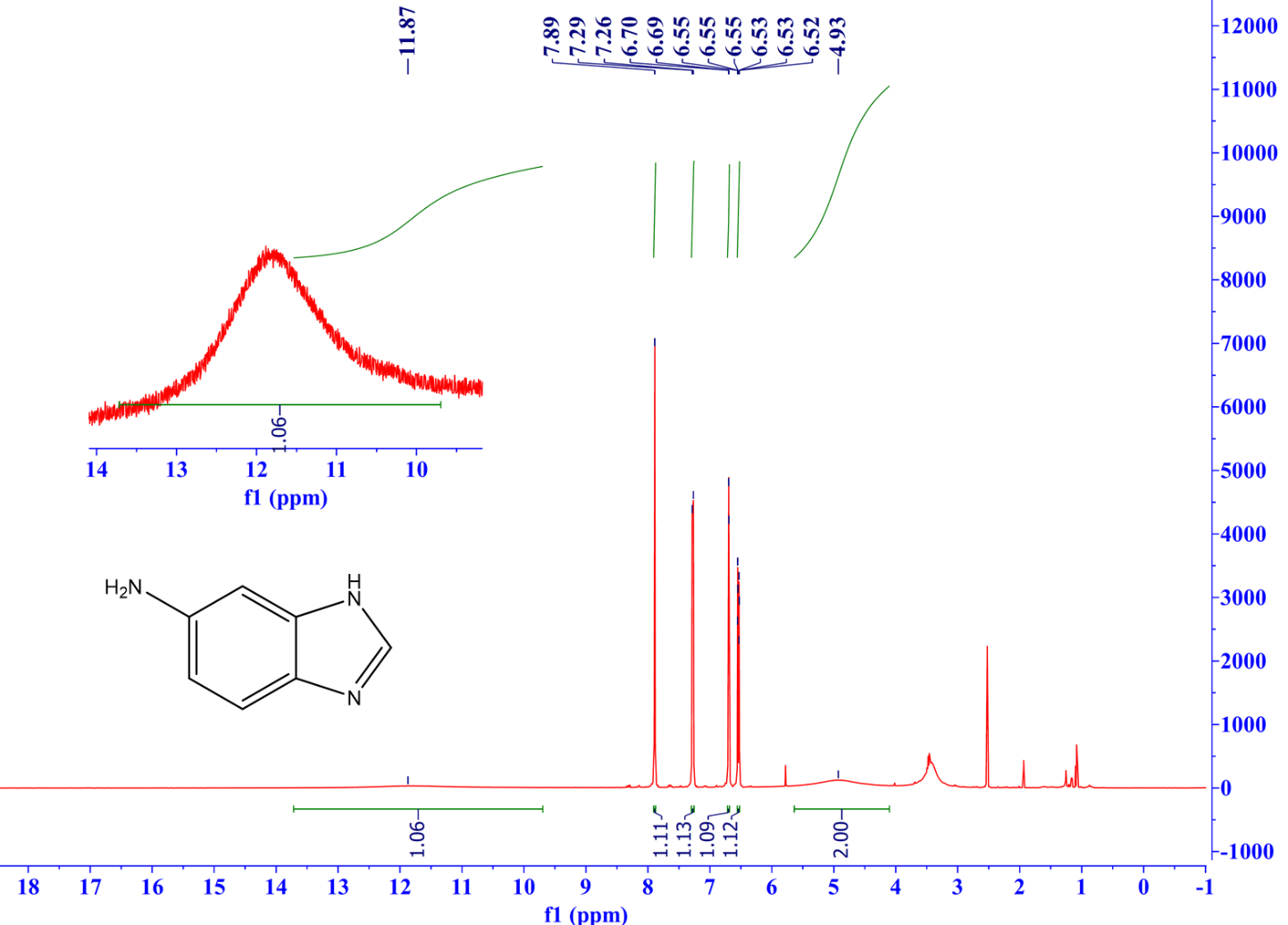


**^1^H NMR of 5-Aminobenzimidazole**

*
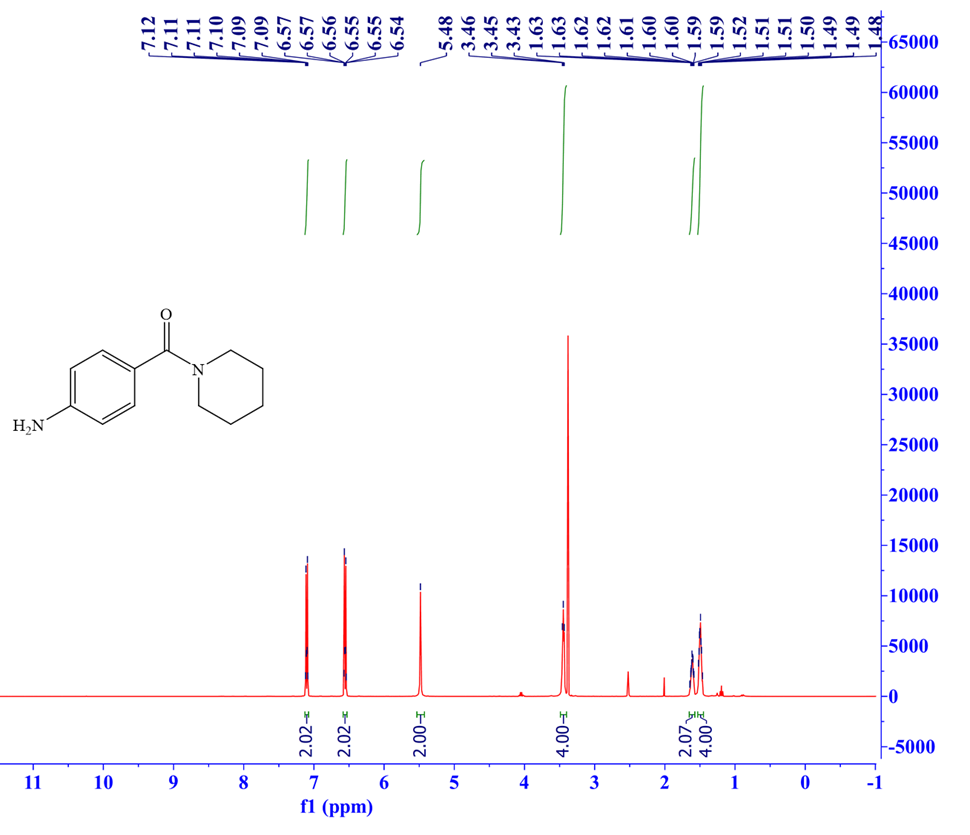
*

**^1^H NMR of (4-Aminophenyl)(piperidin-1-yl)methanone**

*
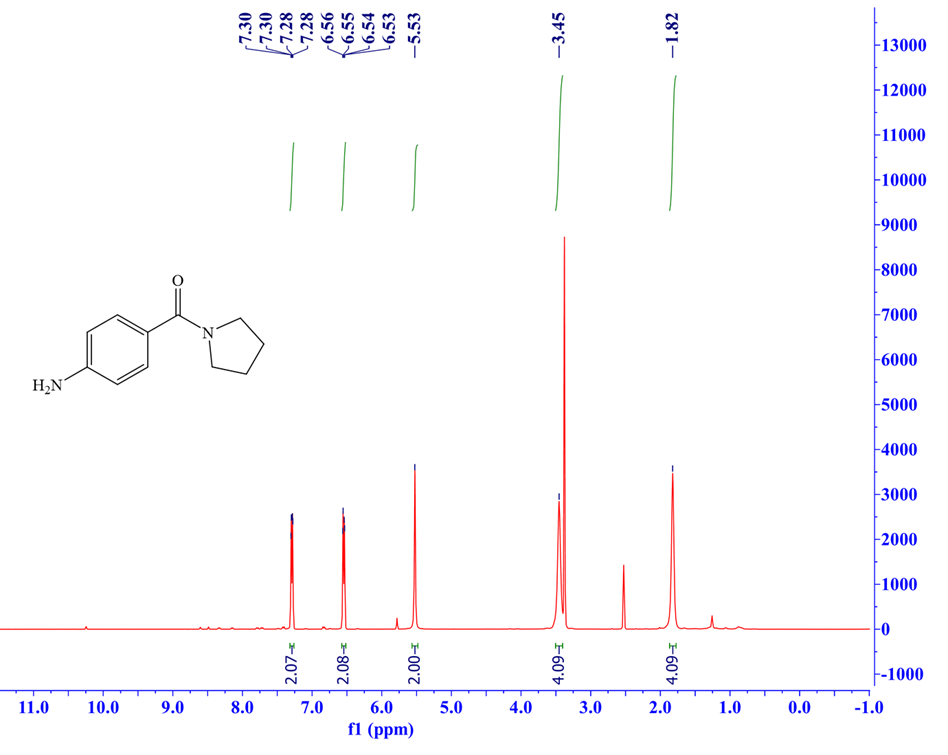
*

**^1^H NMR of (4-Aminophenyl)(pyrrolidin-1-yl)methanone**


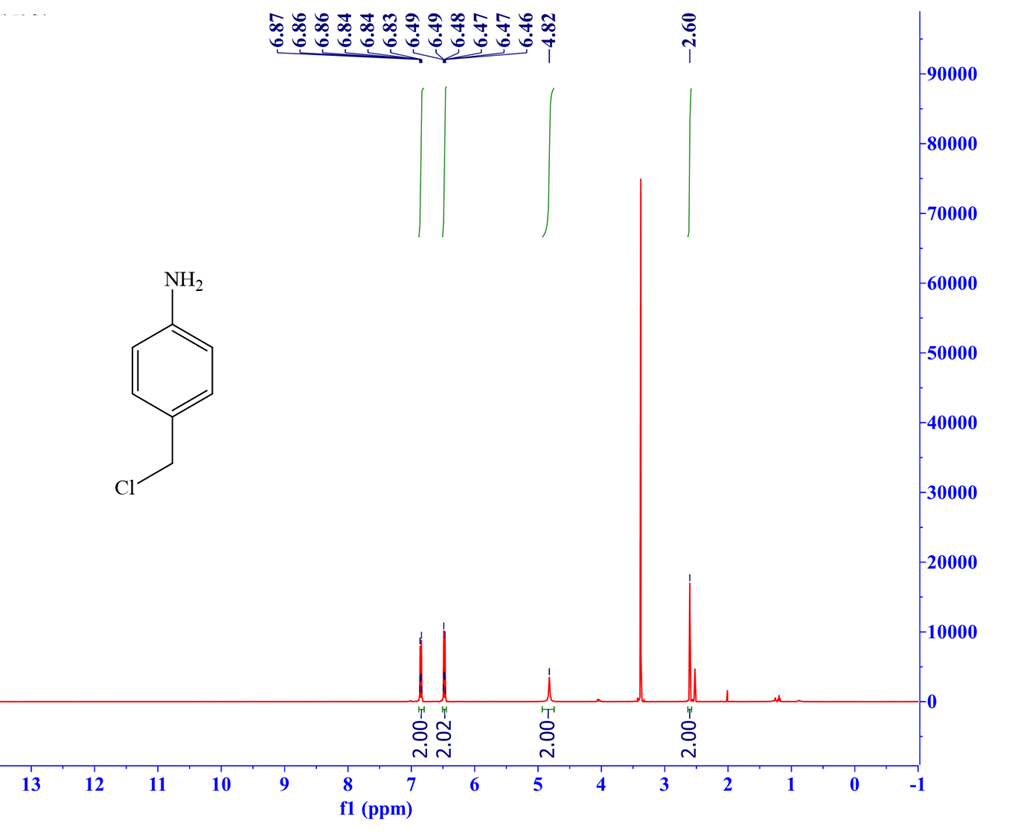


**^1^H NMR of 4-(Chloromethyl)aniline**

*
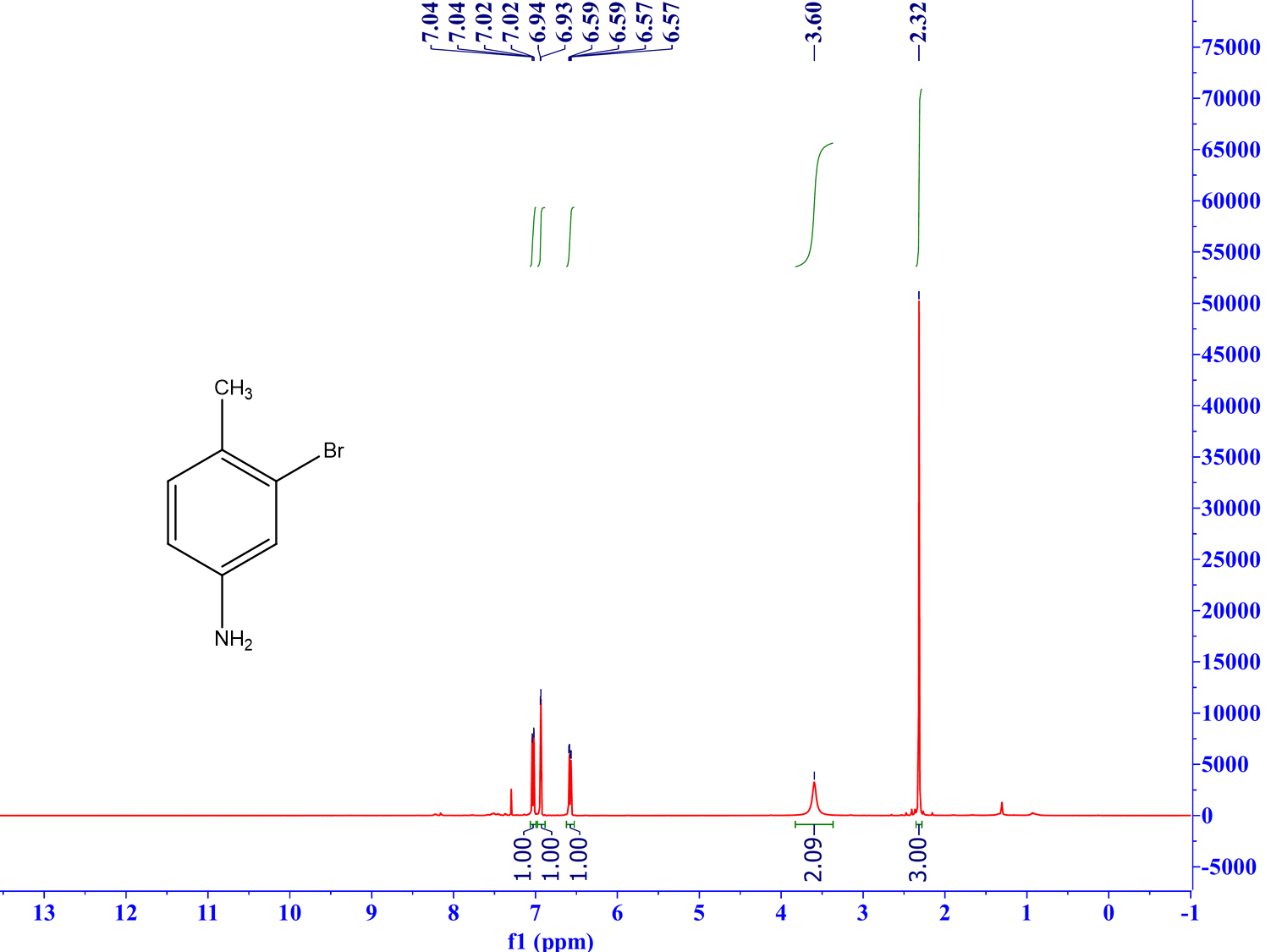
*

**^1^H NMR of 3-Bromo-4-methylaniline**


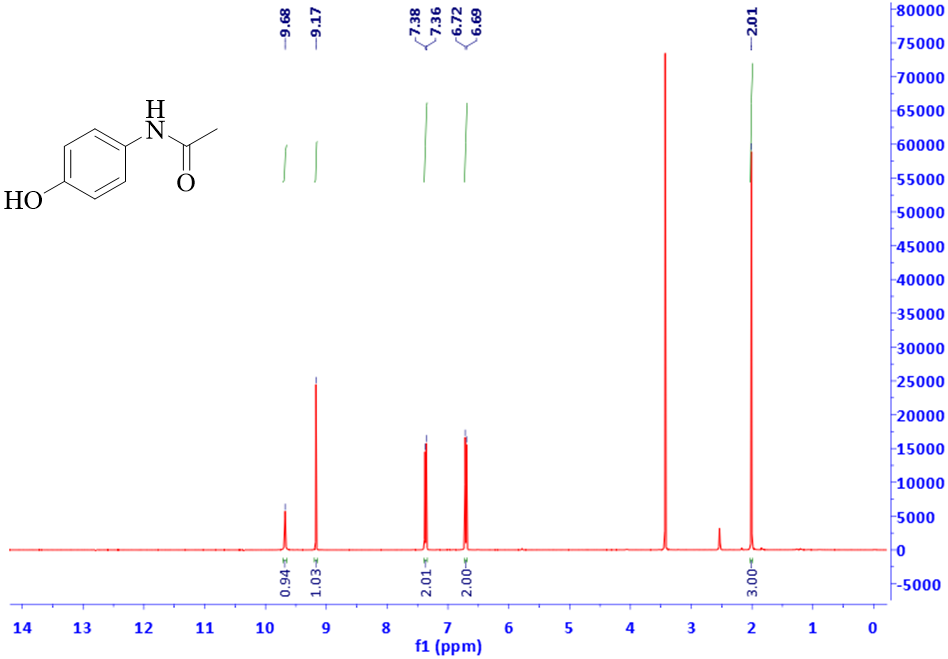


**^1^H NMR of** **Acetaminophen**

**References:**

[1] Jang, M., Lim, T., Park, B. Y., & Han, M. S. Metal-free, rapid, and highly chemoselective reduction of aromatic nitro compounds at room temperature. *J. Org. Chem.* **87**, 910-919, <https://doi.org/10.1021/acs.joc.1c01431> (2022).

[2] Moran, M. J., Martina, K., Baricco, F., Tagliapietra, S., Manzoli, M., & Cravotto, G. Tuneable copper catalysed transfer hydrogenation of nitrobenzenes to aniline or azo derivatives. *Adv. Synth. Catal.* **362**, 2689-2700, <https://doi.org/10.1002/adsc.202000127> (2020).

[3] Niknam, K., Kiasat, A. R., Kazemi, F., & Hossieni, A. Efficient reduction of nitroarenes to the corresponding anilines with sulfur in basic media under solvent-free conditions. *Phosphorus Sulfur Silicon Relat. Elem.* **178**, 1385-1389, <https://doi.org/10.1080/10426500307905> (2003).

[4] Pang, H., Gallou, F., Sohn, H., Camacho-Bunquin, J., Delferro, M., & Lipshutz, B. H. Synergistic effects in Fe nanoparticles doped with ppm levels of (Pd+ Ni). A new catalyst for sustainable nitro group reductions. *Green Chem.* **20**, 130-135, <https://doi.org/10.1039/C7GC02991H> (2018).

[5] Gholinejad, M., Shojafar, M., & Sansano, J. M. Enhanced catalytic activity of natural hematite-supported ppm levels of Pd in nitroarenes reduction. *J. Iran. Chem. Soc.* **17**, 2033-2043, <https://doi.org/10.1007/s13738-020-01908-z> (2020).

[6] Ebadi, M., Asikin-Mijan, N., Md. Jamil, M. S., Iqbal, A., Yousif, E., Md Zain, A. R., & Rahimi Yusop, M. Palladium Nanoparticles on Chitosan-Coated Superparamagnetic Manganese Ferrite: A Biocompatible Heterogeneous Catalyst for Nitroarene Reduction and Allyl Carbamate Deprotection. *Polym.* **15**, 232, <https://doi.org/10.3390/polym15010232> (2023).

[7] Gholinejad, M., Iranpanah, M., Karimi, S., & Sansano, J. M. Cysteine and ionic liquid modified magnetic nanoparticles supported RuCu as a new bimetallic catalyst in reduction reactions. *J. Mol. Struct.* **1298**, 137100, <https://doi.org/10.1016/j.molstruc.2023.137100> (2024).
